# Mechanisms of rapid plant community change from the Miocene Succor Creek flora, Oregon and Idaho (USA)

**DOI:** 10.1371/journal.pone.0312104

**Published:** 2024-11-08

**Authors:** Christopher M. Schiller, Alexander J. Lowe, Thomas A. Dillhoff, Patrick F. Fields, Abigail M. Riley, Ralph E. Taggart, Mark D. Schmitz, Caroline A. E. Strömberg

**Affiliations:** 1 Department of Biology, University of Washington, Seattle, Washington, United States of America; 2 Burke Museum of Natural History and Culture, University of Washington, Seattle, Washington, United States of America; 3 Orma J. Smith Museum of Natural History, College of Idaho, Caldwell, Idaho, United States of America; 4 Department of Earth and Space Sciences, University of Washington, Seattle, Washington, United States of America; 5 Department of Earth and Environmental Sciences, Michigan State University, East Lansing, Michigan, United States of America; 6 Department of Geosciences, Boise State University, Boise, Idaho, United States of America; Sun Yat-Sen University School of Geography and Planning, CHINA

## Abstract

The fossil record of the U.S. Pacific Northwest preserves many Middle Miocene floras with potential for revealing long-term climate-vegetation dynamics during the Miocene Climatic Optimum. However, the possibility of strong, eccentricity-paced climate oscillations and concurrent, intense volcanism may obscure the signature of prevailing, long-term Miocene climate change. To test the hypothesis that volcanic disturbance drove Middle Miocene vegetation dynamics, high-resolution, stratigraphic pollen records and other paleobotanical data from nine localities of the Sucker Creek Formation were combined with sedimentological and geochemical evidence of disturbance within an updated chronostratigraphic framework based on new U-Pb zircon ages from tuffs. The new ages establish a refined, minimum temporal extent of the Sucker Creek Formation, ~15.8 to ~14.8 Ma, and greatly revise the local and regional chronostratigraphic correlations of its dispersed outcrop belt. Our paleoecological analysis at one ~15.52 Ma locality reveals two abrupt shifts in pollen spectra coinciding with the deposition of thick ash-flow tuffs, wherein vegetation dominated by Cupressaceae/Taxaceae, probably representing a *Glyptostrobus oregonensis* swamp, and upland conifers was supplanted by early-successional forests with abundant *Alnus* and *Betula*. Another ephemeral shift from Cupressaceae/Taxaceae swamp taxa in favor of upland conifers *Pinus* and *Tsuga* correlates with a shift from low-Ti shale to high-Ti claystone, suggesting a link between altered surface hydrology and vegetation. In total, three rapid vegetation shifts coincide with ash-flow tuffs and are attributed to volcanic disturbance. Longer-term variability between localities, spanning ~1 Myr of the Miocene Climatic Optimum, is chiefly attributed to eccentricity-paced climate change. Overall, Succor Creek plant associations changed frequently over ≤10^5^ years timespans, reminiscent of Quaternary vegetation records. Succor Creek stratigraphic palynology suggests that numerous and extensive collection of stratigraphically controlled samples is necessary to understand broader vegetation trends through time.

## Introduction

The Miocene was an epoch of dynamic environmental change. Particularly, much attention has been focused on the Miocene Climatic Optimum (MCO, 16.9–14.7 Ma) as a potential analogue for future climate change under intermediate to dire climate scenarios, such as RCP 4.5–6.5 [1], with temperatures ~7°C warmer than today, likely associated with moderately elevated atmospheric pCO_2_ around 400–600 ppm [1–3]. In response to MCO warming, biomes generally advanced poleward with widespread warm-temperate mixed forests at middle latitudes and cool-temperate mixed forests near the Arctic [4, 5]. Superimposed over the long-term pattern of MCO warmth, there is abundant evidence for unusually strong, eccentricity-paced (especially 100 kyr) perturbations of global climate [6, 7] and the carbon cycle [8, 9]. This is hypothesized to be partly due to an Antarctic ice minimum [10], despite a lack of Arctic ice, and eccentricity-paced negative deep ocean carbon sequestration feedbacks [11]. One study suggested that pCO_2_ may have rapidly shifted between 300 and 500 ppm with approximately 100-kyr cyclicity [12]. The impact of these rapid, high-amplitude orbital climate and carbon cycle oscillations on terrestrial ecosystems is unknown.

The Pacific Northwest of the United States also experienced dramatic pulses of volcanism during the MCO [13], which multiple authors have suggested were the primary mechanisms of regional vegetation change, rather than global climate. On short timescales (≤10^4^ years), classic palynological research conducted by Taggart and Cross [14–16] proposed that volcanic disturbance drove vegetation change within short stratigraphic sections of the Middle Miocene Sucker Creek Formation. Over longer timescales (>10^4^ years), Ebinghaus et al. [17] suggested that vegetation on the Columbia Plateau was maintained in a perpetual early- to mid-successional state due to frequent explosive volcanic disturbance based on palynological evidence from interbeds in the Columbia River flood basalts spanning ~16–11 Ma. Indeed, modern, high-resolution geochronology has affirmed that MCO volcanism in the Pacific Northwest was frequent and severe. The main pulse of the Columbia River flood basalts erupted rapidly from 16.7 to 15.9 Ma [18]. Shortly thereafter, ~16.5 Ma, explosive, silicic volcanism began at multiple volcanic centers associated with the migration of the Yellowstone hotspot [19–21]. Further west, western Cascades volcanism, which began in middle Eocene time, continued through the Middle Miocene, although eruptive volumes and rates of uplift may have been relatively quiescent during this period ([22, 23] but see also [24]). A strong signal of volcanic disturbance could potentially overprint signals of MCO climate found within Pacific Northwest paleofloras.

This study aims to test the hypotheses that volcanic disturbance, rather than contemporaneous climate variability, drove MCO plant community composition change in the Pacific Northwest (1) over short timescales (≤10^4^ years, *sensu* Cross and Taggart [14]) and (2) over long timescales (>10^4^ years, *sensu* Ebinghaus et al. [17]). To do so, we employ multiproxy, high-resolution analysis of the Watersnake locality of the Succor Creek flora, reconstructing plant communities (pollen supplemented by macrofossils and phytoliths), volcanic disturbance (lithostratigraphy), and lithological changes in the basin (elemental geochemistry). High-precision U-Pb zircon geochronology of tuffs was performed to provide temporal context for our paleobotanical datasets, permit comparison with contemporaneous climate records, and determine rates of vegetation change.

### The Succor Creek flora

**Fig 1:**
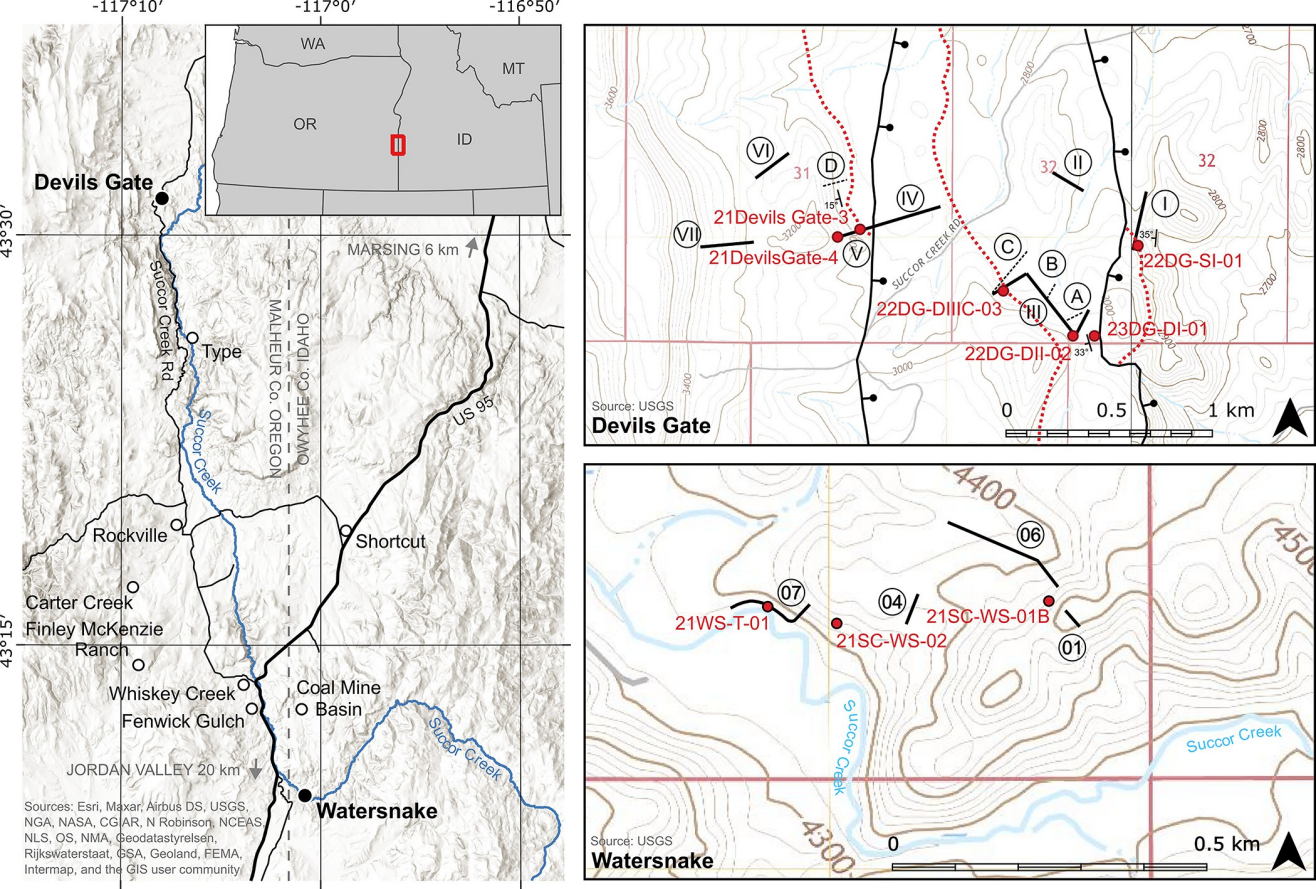
Succor Creek flora locality map. (A) Locations of existing Sucker Creek Formation palynological localities (open dots) and with localities with significant new contributions in this manuscript (black dots). At the Devils Gate locality, stratigraphic sections of Satchell [38] (black, solid) and Downing [37] (black, dotted) are given relative to the locations of tuff samples for U-Pb zircon chronology (red dots) and the location of the Obliterator Ash (red, dotted), used to correlate between fault blocks. At the Watersnake locality, stratigraphic sections (this study, black, solid) are given relative to the location of tuff samples (red dots). Hillshade basemap sourced from Esri and is used herein under license, © Esri, all rights reserved. Topographic basemaps sourced from the U.S. Geological Survey, public domain.

Plant fossils of the Middle Miocene (Langhian) Succor Creek flora (flora name following usage in the literature since Giannasi and Niklas [25]) have been described from the Sucker Creek Formation (formation name following Kittleman et al. [26]) at more than one hundred localities in southeastern Oregon and southwestern Idaho (Fig 1). The Succor Creek macroflora was initially described by Knowlton (*in* Lindgren [27]) and has been subsequently revised by several workers [28–31] culminating in the most recent, comprehensive efforts by Graham [32, 33] and Fields [34]. A palynoflora was also described by Graham [32, 33] and added to by Taggart [35]. A megafauna has been reported [36, 37], though animal fossils are not typically found in the same facies as the fossil flora.

The Sucker Creek Formation consists of interbedded sedimentary sequences of lacustrine, fluvial, and paleosol origin and volcaniclastic deposits [26, 39]. The long-standing observation has been that the lacustrine facies are more typical of the lower part of the formation, while fluvial and paleosol facies are more common in the upper part [19, 37]. Although the Sucker Creek Formation is extensively exposed in the Owyhee region of Oregon and Idaho, particularly along Succor Creek itself, attempts at correlation between spatially separated sections have generally been unsuccessful (but see also Taggart [40]). The contemporaneous Lake Owyhee Volcanic Field lies immediately to the west of the formation, populating the sediments with several tuffs, notably the Tuff of Leslie Gulch [19]. The proximity of the Lake Owyhee Volcanic Field makes the Sucker Creek Formation an ideal area to study volcanic disturbance where disturbance was likely frequent and severe. Intermediate lava flows interbedded with Sucker Creek Formation lake sediments have been used as evidence that the lake basin(s) was created by lava flow dams [19]. Existing ^40^K/^40^Ar and ^40^Ar/^39^Ar ages generally place the Sucker Creek Formation between 16 and 15 Ma (S1 Table).

The vegetation of the Succor Creek flora varies across localities, but *Quercus*, *Cedrela*, *Acer*, and *Platanus* are usually common as macrofossils while *Ulmus*, *Picea*, *Quercus*, Cupressaceae/Taxaceae, and *Tsuga* are common palynomorphs [33, 34]. Nested within this scheme, stratigraphic palynology from several localities demonstrates considerable vegetation variation within and between localities. At the Rockville and Shortcut localities, initial forested mosaics of lowland hardwoods (chiefly *Ulmus*/*Zelkova*, *Alnus*) and montane conifers (chiefly *Picea*) declined, in favor of an early successional community (including *Pinus*, Asteraceae, Malvaceae, Amaranthaceae) [40]. At the Devils Gate locality, a 65-m thick palynological section showed several cycles of hardwood-conifer forest mosaic shifting to an *Ulmus*-dominated forest, which was attributed to rapid climate change, fire, or other forms of disturbance [38]. Additional, discontinuous pollen spectra through ostensibly younger, largely volcaniclastic units at Devils Gate are abundant in early successional shrubs and herbs, possibly the product of volcanic disturbance [38]. Volcanic disturbance has been the favored, hypothesized mechanism of vegetation change within Succor Creek sections [14–16]. However, volcanic disturbance inferred from pollen data has not been tied to independent records of disturbance (i.e., presence of tuffs) and mechanisms of vegetation change in some sections remain mysterious [38].

## Methods

### Field sampling and stratigraphy

The Watersnake locality of the Succor Creek flora (hereafter simply “Watersnake”) of Fields [34] was selected for its apparently long, stratigraphically continuous section of lacustrine mudrocks interbedded with multiple tuffs. Watersnake (43.2°N, 117.0°W, 1300 m.a.s.l.) is a prominent exposure of the Sucker Creek Formation, situated along a bend of Succor Creek near the junction of US 95 and Old US 95 (Fig 1). Modern vegetation in the Watersnake vicinity consists of *Artemisia tridentata* steppe with *Dasiphora fruticosa* and *Chrysothamnus viscidiflorus*, as well as herbs, specifically Poaceae (abundant *Bromus tectorum*), Amaranthaceae, *Eriogonum*, and *Rumex*. *Salix*, Poaceae, *Carex*, and *Juncus* grow along Succor Creek. We measured a composite stratigraphic section at Watersnake and described sedimentology at the sub-meter scale. ~30-g rock samples were collected for elemental geochemistry, pollen, and phytoliths every 20 cm through the section, except through two thick volcanic ash-flow tuffs which were assumed to have poor pollen preservation. Additional pollen samples were collected immediately above and below each tuff to assess potential ecological impacts of each eruption. ~2-kg tuff samples were collected from each tuff for U-Pb zircon geochronology. Plant macrofossils were collected from lacustrine shales near the Watersnake locality across a 2.8 m stratigraphic interval (~2.2–5.0 m composite section), though most intensively from a 1.5 m stratigraphic interval (~3.5–5 m composite section). Collections were made from four quarries laterally spanning 50 m to account for spatial variability. To reliably calculate relative abundance and reconstruct diversity, a census collection technique was employed, whereby all fossil plant material assignable to a morphotype was collected or, for more poorly preserved specimens of common morphotypes, photographed in the field.

Further field investigations were conducted at classic Succor Creek localities such that existing palynological data could be leveraged in the light of new U-Pb zircon geochronology. The Devils Gate locality was selected for its apparently long stratigraphic interval in the northern outcrop belt of the Sucker Creek Formation (Fig 1) and for its well-documented palynological records [38]. Fault repetition of the stratigraphy at Devils Gate was noted in previous studies [34, 37–39]. To support our stratigraphic investigations and test hypotheses regarding the correlation of volcanic horizons in repeated sections across fault blocks, we located and remeasured existing sections [37, 38]. Named tuff beds were sampled for U-Pb zircon analyses, including I through IV of Downing [37] and Downing and Swisher [41], plus the prominent ‘silver ash’ in the upper portion of Satchell’s [38] Unit I. Stratigraphic logs as well as contact tracing and analysis of bedding attitudes were used to identify the positions of N-S striking, down-to-the-east normal faults transecting the study area (Fig 1). The Rockville locality (Fig 1) was similarly selected for its extensive palynological records [40]. A prominent white tuff indicated by Taggart [40] was sampled for U-Pb zircon analysis.

Field work was conducted under Idaho Bureau of Land Management Paleontological Resources Use Permit IDI-39267 and Oregon/Washington Bureau of Land Management Casual Use Permit Schiller2022-004. Additional field work was conducted, with permission, on private property in the Succor Creek area of Idaho and Oregon.

### Laboratory analyses

#### Elemental geochemistry

Elemental geochemistry from X-ray fluorescence (XRF) is a well-established proxy for siliciclastic deposition in fine-grained sediments from late Quaternary lake sediment cores [42, 43]. Here, we used a similar methodology to interpret watershed dynamics in the Watersnake section. From all available samples, small ~2-3-g subsamples were thoroughly air dried and ground to a fine powder using a mortar and pestle to ensure homogeneity. Ground samples were placed in plastic X-ray cells, covered with 4-μm Ultralene^®^ film, and analyzed using a Bruker Tracer III-V^®^ portable XRF instrument. The instrument was configured with a 25-μm Ti filter, an external vacuum pump, and stimulation parameters of 40 keV, 4 μA, and 1-minute counting duration. Samples were measured three times with a 120° rotation between each measurement to further account for sample heterogeneity. Elemental counts were enumerated by Bayesian deconvolution using Bruker S1PXRF^®^ software and counts were modelled using Bruker ARTAX^®^ software. Averaged sample counts were empirically calibrated to ppm using a set of 10 standards and an empty X-ray cell (S2 Table). Abundance of titanium is used herein as a proxy for siliciclastic, as opposed to biogenic, input to the lake [42, 43].

## U-Pb geochronology

Volcanic tuffs were dated via U-Pb zircon geochronology using the chemical abrasion isotope dilution thermal ionization mass spectrometry (CA-IDTIMS) technique. Abundant populations of equant to elongate prismatic zircon crystals, approximately 100–300 μm in long dimension, were separated from disaggregated clay-rich tuff samples with an ultrasonic clay separator [44], followed by conventional density and magnetic methods. The entire zircon separate was placed in a muffle furnace at 900°C for 60 hours in quartz beakers to anneal minor radiation damage, preparing the crystals for subsequent chemical abrasion [45]. Following annealing, individual sharply faceted, high aspect ratio grains with axial melt inclusions indicative of rapid subvolcanic growth were hand-picked for isotopic analysis. The methods for CA-IDTIMS U-Pb zircon geochronology followed those published by Macdonald et al. [46]. Zircon crystals were subjected to a modified version of the chemical abrasion method of Mattinson [45], whereby single crystals were individually abraded in a single step with concentrated HF at 190°C for 12 hours. The remaining residual crystals were thoroughly rinsed before spiking with the ET535 tracer [47, 48], with complete dissolution at 220°C for 48 hours, followed by ion chromatographic purification of U and Pb [49] and isotope ratio analysis by thermal ionization mass spectrometry [50].

U-Pb dates and uncertainties for each analysis were calculated using the algorithms of Schmitz & Schoene [51], the U decay constants of Jaffey et al. [52], and the natural ^238^U/^235^U ratio estimate from Hiess et al. [53]. Quoted errors for calculated weighted means are in the form ±X(Y)[Z], where X is solely analytical uncertainty; Y is the combined analytical and tracer uncertainty; and Z is the combined analytical, tracer and ^238^U decay constant uncertainty. Weighted mean ages are established from the youngest group of concordant ^206^Pb/^238^U dates that pass the modified Thomson Tau outlier rejection criteria (p = 0.05, [54]). These ages are interpreted to represent the time since eruption and deposition. Individual zircon dates that are older than the youngest statistical group are interpreted to reflect magmatic antecrysts or epiclastic inheritance.

At the Watersnake locality, an age-depth model was established to constrain rates of vegetation and landscape change. A Bayesian age-depth model was developed from U-Pb ages using Bacon software [55]. *A priori* assignment of mean sediment accumulation rate was set to 500 yr/m. It was assumed that none of the section strata were deposited “instantaneously”, including the thick tuffs. Hence, the entirety of the measured section was included in the model.

## Pollen and phytoliths

Pollen was extracted from a subset of mudrock samples at approximately 1-m intervals, plus samples stratigraphically above and below volcaniclastic deposits to assess the potential impact of volcanic eruptions on vegetation. The samples were thoroughly washed, crushed to mm-size, and massed to 3 ± 0.1 g. Pollen extraction followed standard procedures [56], including treatments with HCl, HF, KOH, acetolysis, and sieving (180 and 7 μm meshes). Residues were preserved in t-BuOH and mounted in silicone oil. A minimum of 300 identifiable, terrestrial pollen grains were identified at 400× magnification or greater and assigned to extant taxa based on comparison with relevant atlases [57, 58], plates from publications of regional Miocene palynofloras [33, 40, 59–61], and the Jane Gray modern reference collection in the Burke Museum of Natural History and Culture, University of Washington (UWBM). Pollen grains were assigned to extant families, genera, or pollen type based on the co-occurrence of those extant taxa in the macroflora of Succor Creek flora where possible. Terrestrial pollen data are expressed as percentages of the terrestrial pollen sum and other palynomorphs are expressed as percentages of the sum of all palynomorphs. Spectra were separated into zones using stratigraphically constrained cluster analysis (CONISS, [62]) based on an increase in the sum of squares between clusters (ISS) > 0.4.

12 sediment samples in the Watersnake section were processed for phytoliths and other biosilica following standard protocols [63–65], which included treatment with HCl, Schultze’s solution (HNO_3_ + KClO_3_), and ZnBr_2_ heavy liquid. Biosilica yield was mounted in Cargille™ Meltmount for permanent storage and viewed under 1,000× magnification. Phytolith morphotypes were classified using previous publications [65, 66] and the UWBM phytolith reference collection into phytolith functional types (PFT) categories (e.g., forest indicators, pooid grasses). Vegetation type and structure were reconstructed by comparing the relative abundance composition of phytolith assemblages focusing on diagnostic PFTs (forest indicators, open- and closed-habitat grasses) [64, 66]. Specifically, the relative abundance of diagnostic grass phytoliths is used to indicate the importance of grasses in the environment; the composition of forest indicator morphotypes helps understand the broad composition of silica-producing trees and shrubs (i.e., dicots vs. conifers), and the composition of grass phytolith assemblages help reconstruct the relative abundance of different open-habitat taxa (C_3_ vs. C_4_, cool-adapted Pooideae vs. warm-adapted so-called PACMAD grasses) and closed-habitat grasses.

## Plant macrofossils

Collected and field*-*photographed plant macrofossils were assigned to morphotypes utilizing leaf architectural traits [67] and compared with regional monographs [31, 33, 34] and previous collections housed at the Orma J. Smith Museum of Natural History, College of Idaho. Macrofossils of differing organs (e.g., foliar, reproductive) were assigned to separate morphotypes even if presumed to be from the same species. Isolated branchlets of needle- and scale-leaved conifers (e.g., *Glyptostrobus oregonensis*) and individual leaflets of compound leaves (e.g., Fabaceae) were counted as single specimens. Relative abundances of foliar morphotypes were calculated as a percentage of total foliage and of total non-monocot angiosperm foliage. The latter accounts for potential differences in taphonomic processes between gymnosperms, monocot angiosperms, and non-monocot angiosperms. The abundances of various reproductive structures were reported as counts rather than percentages. Charcoal was studied through thin section microscopy at 200× magnification to observe cellular detail and attribute to taxon.

### Synthesis of existing palynological data

Pollen data from existing studies of the Succor Creek flora (Fig 1) have been digitized, where available. Pollen count data from the Carter Creek, McKenzie Ranch, and Fenwick Gulch localities [33] were digitized from published tables. In the absence of surviving count data, percentage data were digitized from high-resolution scans of taxon-specific pollen diagrams from the Devils Gate [38], Rockville, Shortcut, and Whiskey Creek [40] localities. We were unable to locate materials, data, or taxon-specific pollen diagrams associated with work done at the Type [14, 68] and Coal Mine Basin (Taggart *in* Walden [69]) localities. Reported pollen types were updated to modern taxonomic nomenclature and harmonized.

To test whether vegetation changes within each locality were linked to volcanic disturbance, we compared pollen spectra with independent evidence of a volcanic disturbance mechanism, chiefly the presence of primary tuffs in lithostratigraphic section. Units “2”, “3”, and “4” of Devils Gate, McKenzie Ranch, Carter Creek, and Fenwick Ranch were excluded due to a lack of dense stratigraphic sampling. To ensure that the changes in pollen spectra were uniformly assessed in all sections, CONISS [62] was used with the same arbitrary threshold used to describe zones in the Watersnake section (increase in sum of squares, ISS > 0.4). To visualize differences in pollen spectra between localities in ordination space, we employed non-metric multidimensional scaling (NMDS) with the Bray-Curtis dissimilarity metric and a square-root transformation of the pollen percentage data. Preliminary analysis suggested that rare taxa (<1% at all stratigraphic levels) were highly influential on the ordination analysis. Rare taxa were subsequently dropped, since most of these taxa were only identified by a single analyst and were unlikely to reflect real differences in diversity between sites.

## Results

### Stratigraphy and geochemistry

#### Watersnake locality

**Fig 2:**
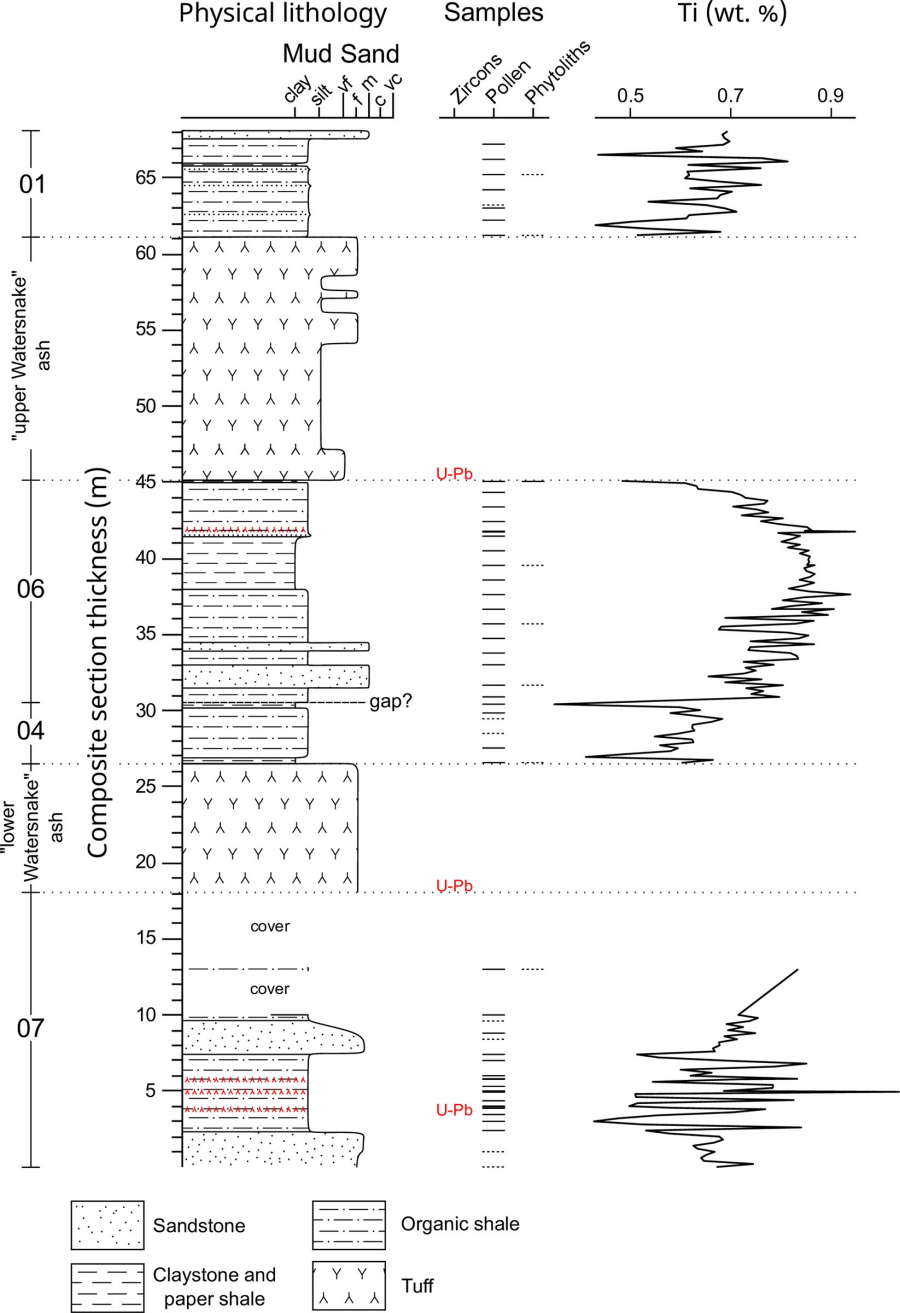
Watersnake locality stratigraphy. Stratigraphic lithology, sampling horizons (productive—solid lines, poorly preserved—dotted lines), and total sediment titanium content (wt. %) through the Watersnake section. Increased Ti content of sediment suggests increased siliciclastic deposition.

Watersnake locality lithostratigraphy consists of approximately 68 m of mudrocks, arkosic sandstones, and vitric airfall and ash-flow tuffs, divided here into the (from bottom to top): “lower lacustrine unit”, “lower Watersnake ash-flow tuff”, “middle lacustrine unit”, “upper Watersnake ash-flow tuff”, and “upper lacustrine unit” (Fig 2). Several basaltic dikes intrude the Sucker Creek Formation in the study area as well. This stratigraphy established in measured sections within a single fault block at Watersnake appears to be repeated at exposures further west and south of Succor Creek, but faulting and slumping makes direct correlation difficult. Watersnake stratigraphically is probably older than the nearby Coal Mine Basin and “Arrowhead” sections described by Walden [69] based upon regional bedding attitudes and have an unknown stratigraphic relationship with other Sucker Creek localities. We consider comprehensive resolution of the stratigraphic complexities within these southern exposures of the Sucker Creek Formation, although badly needed, outside the scope of this study.

Mudrocks at Watersnake consist primarily of brownish-gray, tuffaceous shales with arkosic clasts (Fig 2). These shales are particularly distinctly laminated in the lower lacustrine unit and become more poorly laminated and sandy up section. In addition, purplish-brown paper shales are found in isolated stratigraphic intervals in the middle and upper lacustrine units but appear to rapidly pinch out laterally. Gray claystone also occurs in the middle lacustrine unit. Except in the claystone, fossil leaves and plant hash are common throughout the mudrocks studied. Dark gray to brown, massive medium-grained wackes are found interbedded with mudrocks throughout the measured section (Fig 2) and include abundant subangular lithics, muscovite, potassium feldspar, and plagioclase in thin section. The immature, granitic composition of the wackes suggests a local, crystalline source, such as the Idaho [26] or the Silver City [14] batholiths. The massive, poorly sorted texture of the wackes would be consistent with deposition through turbidite flows, rather than beach or fluvial environments. Ti concentrations throughout the sediments are highly variable, probably owing to variable siliciclastic sediment input. However, paper shales are associated with distinctly low Ti concentrations, while the claystone is associated with consistently high Ti (Fig 2).

Four thin (up to 4 cm thick) normally-graded bentonite layers are noted within the lower and middle lacustrine units and are interpreted as airfall tuffs. Of these, only the lowest bentonite (+4 m above the base of the section illustrated in Fig 2) yielded zircon crystals of volcanic origin. The lower Watersnake ash-flow tuff (+18 to 26.5 m above base) consists of 8.5 m of white, bedded to laminated, fine to medium grained originally vitric ash, largely altered to zeolitized clay throughout. Large (up to 8 cm diameter), silicified charcoal fragments were found as float below the ash but were not observed *in situ*. The base of the lower Watersnake ash-flow tuff is poorly exposed but was excavated for sampling for U-Pb geochronology. Thickness, sorting, and layering support its characterization as an ash-flow tuff deposit. The upper Watersnake ash-flow tuff (+45 to 61 m above base) consists of 16 m of white, bedded, normally graded, fine to medium grained vitric ash and lapilli, altered to clay in many intervals. Mudrock clasts (some >10 cm in diameter) occur near the base and abundant woody plant fragments, some apparently pyrolyzed, occur except near the base. The thickness of the unit, presence of mudrock clasts (interpreted as rip-up clasts), and pyrolyzed wood suggest an ash-flow deposit with a basal airfall vitric tuff layer.

#### Devils Gate locality

At the Devils Gate locality, as studied by Satchell [38] and Downing [37], the Sucker Creek Formation is exposed below the overlying Pliocene Jump Creek Rhyolite in a series of east-northeast facing exposures separated by N-S trending down-to-the-east normal faults [70]. Bedding in these fault blocks dips gently to moderately (<35°) to the west-southwest. Satchell [38] measured and described a series of seven stratigraphic sections totaling over 735 m including covered intervals, which were stacked into a composite section on the basis of lithological and macrofossil correlation (Fig 1). Downing [37] created a 240-m-thick composite section from four measured sections (A-D) on the east and west sides of Succor Creek Road, using four prominent volcanic tuffs and a distinctive horizon of in situ tree stumps as correlation markers (Fig 1). The most conspicuous marker horizons in the section are volcanic tuffs, including Downing’s Ash III, otherwise referred to as the ‘Obliterator Ash’ [41]. Recognizing two N-S trending normal faults with down-to-the-east displacement through our own field mapping and interpretation of aerial imagery, we utilized horizons of the Obliterator Ash across the study area (dotted line; Fig 1), to reliably correlate across a pair of down-to-the-east normal faults that repeat the stratigraphic section. The identity of the Obliterator Ash in each fault block was confirmed by direct radioisotope dating.

Our mapping supports the general interpretative stratigraphic framework of Downing [37], in which his sections A, B, and C below the Obliterator Ash and east of Succor Creek Road reside on the hanging wall block of a normal fault running N-S to the west of Succor Creek Road, whereas section D is preserved on the footwall block of the same fault above a repetition of the Obliterator Ash (Fig 1). By contrast, our mapping documents that several of the sections of Satchell [38] are fault repetitions. We interpret her sections I and III as repeated sections below the Obliterator Ash, and sections IV and V as repeated sections above the Obliterator Ash (Fig 1).

### U-Pb geochronology

#### Rockville locality

Our sample of the ‘white ash bed’ of Taggart [40] provided an abundant, high-quality sampling of large, elongate, prismatic zircon crystals (Table 1, S3 Table). Five of six crystals analyzed by CA-IDTIMS produced concordant and equivalent isotopic data with a weighted mean ^206^Pb/^238^U date of 15.782 ± 0.013(0.013)[0.022] Ma (MSWD = 1.65), which we interpret as the eruption and depositional age of the volcanic event. A single crystal produced a date ~0.5 Myr older than this group and is interpreted as xenocrystic (epiclastic volcanic) and unrelated to penultimate magmatic differentiation and eruption.

**Table 1:** Summary of Sucker Creek Formation CA-IDTIMS U-Pb ages^1^.

Sample	Wt. mean ^206^Pb/^238^U ages	Equivalents
**Devils Gate Locality**		
21Devils Gate 3	14.802 ± 0.026 Ma	**‘Obliterator Ash’** [41], Ash III [37]
22DG-IIIC-03	14.798 ± 0.027 Ma	
22DG-SI-01	14.801 ± 0.010 Ma	‘silver ash’ [38]
*ensemble ‘Obliterator Ash’*	*14*.*799 ± 0*.*007 Ma*	
22DG-DII-02	*15*.*120 ± 0*.*031 Ma*	**‘Loggers Ash’** [41], Ash II [37], locality of the ‘saddle stump horizon’ [38]
**Watersnake Locality**		
21SC-WS-01B	*15*.*512 ± 0*.*022 Ma*	
21SC-WS-02	*15*.*512 ± 0*.*025 Ma*	
21WS-T-01	*15*.*546 ± 0*.*018 Ma*	
**Rockville Locality**		
21Rockville ash	*15*.*782 ± 0*.*013 Ma*	‘white ash bed’ [40]

^1^Italicized where used in final age determinations and age-depth models

## Watersnake locality

Three volcanic tuffs from the Watersnake locality yielded precise and accurate eruption and deposition ages, including one thin bentonite from the lower lacustrine unit and the two thick ash flow tuffs that divide the section into units (Table 1, S3 Table). Sample 21WS-T-01 was the lowest sampled thin bentonite from the base of the macrofloral census layers and provided a sparse but high-quality group of prismatic zircon crystals. Five of eight crystals analyzed by CA-IDTIMS produced concordant and equivalent isotopic data with a weighted mean ^206^Pb/^238^U date of 15.546 ± 0.018(0.019)[0.025] Ma (MSWD = 0.71). Three additional crystals produced dates ~0.1 Myr older than this group and are interpreted as either epiclastic or antecrystic contributions to the crystal cargo, unrelated to penultimate magmatic differentiation and eruption. The thick lower ash-flow tuff at the Watersnake locality yielded a weighted mean ^206^Pb/^238^U date of 15.512 ± 0.025(0.026)[0.031] Ma (MSWD = 0.13) from the five youngest crystals, with four crystals yielding older Miocene to Cretaceous dates. Similarly, the upper ash-flow tuff at the Watersnake locale also yielded a weighted mean ^206^Pb/^238^U date of 15.512 ± 0.022(0.023)[0.028] Ma (MSWD = 0.13) from the six youngest crystals, with two older xenocrysts.

**Fig 3:**
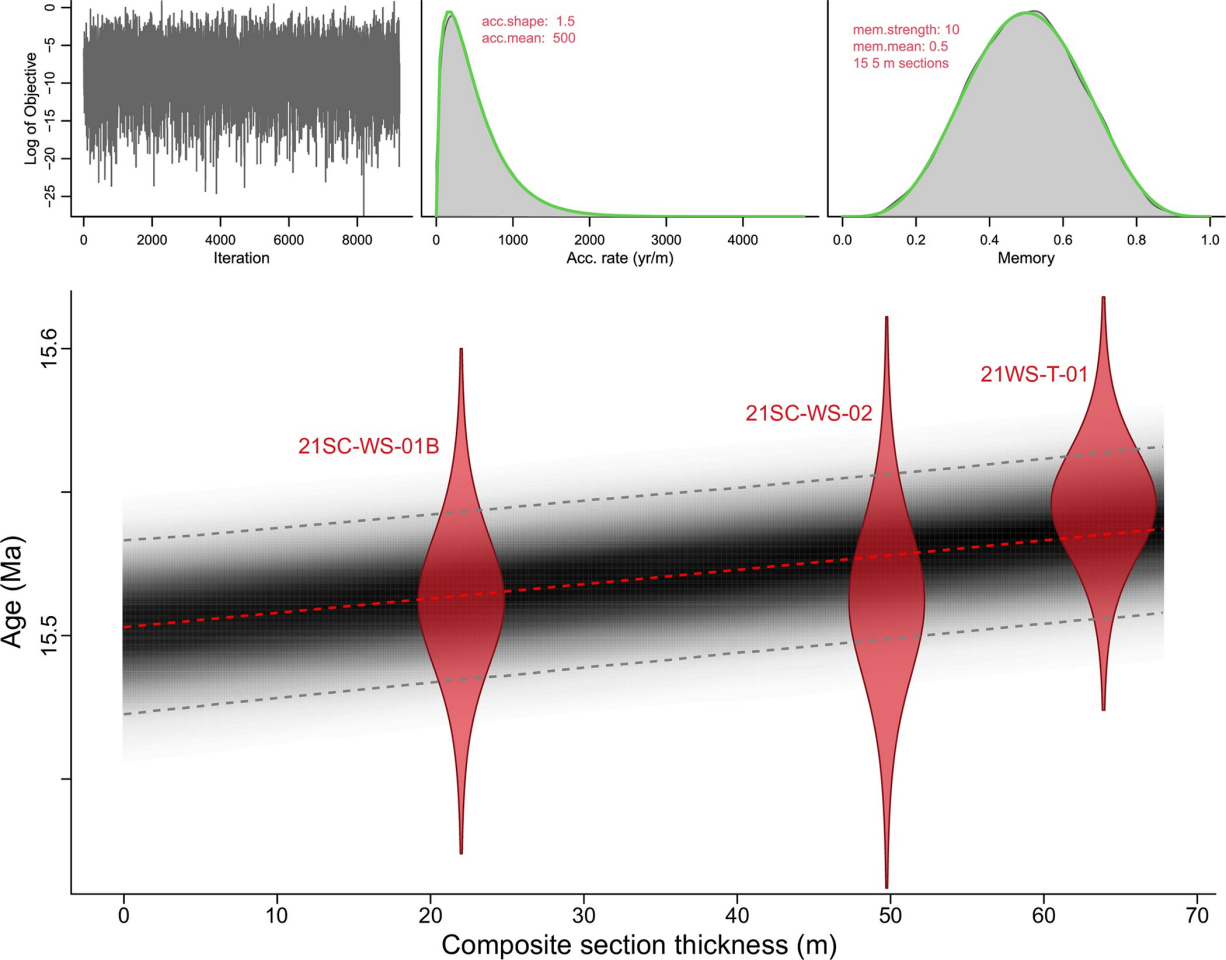
Watersnake locality Bacon age-depth model. Probability distributions are plotted for each U-Pb age determination (red silhouettes). Red dashed line is the median probability age from all run age-depth iterations, representing the best point estimate of age for any given depth. Gray point cloud represents age model probability and contains a 95% confidence interval (dashed gray lines). Iteration history (left top), prior and posterior densities of the mean accumulation rate (middle top), and prior and posterior of the memory (right top) suggest reasonable adherence of the model to a priori mean accumulation rate and memory assignments.

Due to the close temporal spacing of the Watersnake tuffs, the resultant age model (Fig 3) indicates a rapid sedimentation rate. Extrapolated median probability ages at the base of the section, 15.537 (95% probability 15.566–15.508) Ma, and the top of the section 15.503 (95% probability 15.533–15.473) Ma, suggest a duration of 34 kyr for the section and rate of ~500 yr/m.

## Devils Gate locality

Four volcanic tuff samples from the Devils Gate locale yielded precise and accurate eruption and depositional ages (Table 1, S3 Table). Downing’s Ash I (Lough Ash) and Ash IV (unnamed) were also sampled in this study but had very poor yields of zircon crystals too small for precise geochronology.

Downing’s Ash II or the ‘Loggers Ash’, collected proximal to a *Glystostrobus* stump bed in the central fault block of the study area, yielded an abundant sampling of elongate prismatic zircon crystals. Four of six crystals analyzed by CA-IDTIMS produced concordant and equivalent isotopic data with a weighted mean ^206^Pb/^238^U date of 15.120 ± 0.031(0.032)[0.036] Ma (MSWD = 0.72). Two additional crystals produced dates resolvably older than this group and are interpreted as either epiclastic or antecrystic contributions to the crystal cargo, unrelated to penultimate magmatic differentiation and eruption.

Three samples of Downing’s Ash III or the ‘Obliterator Ash’ collected across the three interpreted fault blocks of the study area yielded similar zircon populations, and identical U-Pb ages within their uncertainties. Sample 21DevilsGate-3 was collected from the westernmost fault block near the base of Downing’s section D-D’ and Satchell’s section V, and yielded a weighted mean ^206^Pb/^238^U date of 14.802 ± 0.026(0.026)[0.031] Ma (MSWD = 0.72; n = 4). Sample 22DG-DIIIC-03 was collected from the central fault block stratigraphically above the Loggers Ash and near the top of Downing’s section C-C’ and Satchell’s section III. Four of five crystals analyzed by CA-IDTIMS produced concordant and equivalent isotopic data with a weighted mean ^206^Pb/^238^U date of 14.797 ± 0.027(0.027)[0.031] Ma (MSWD = 1.56), with one significantly older xenocryst. Sample 22DG-SI-01, the ‘silver ash’ of Satchell [38] on the easternmost fault block at the top of Unit I yielded a weighted mean ^206^Pb/^238^U date of 14.801 ± 0.010(0.011)[0.019] Ma (MSWD = 0.45; n = 6) with two slightly older antecrysts. An ensemble age for the Obliterator Ash calculated from all concordant and equivalent zircon crystals across the three samples is 14.799 ± 0.007 (0.009) [0.018] Ma (2s); MSWD = 0.42 (n = 12).

## Watersnake pollen, phytoliths, and macrofossils

**Fig 4:**
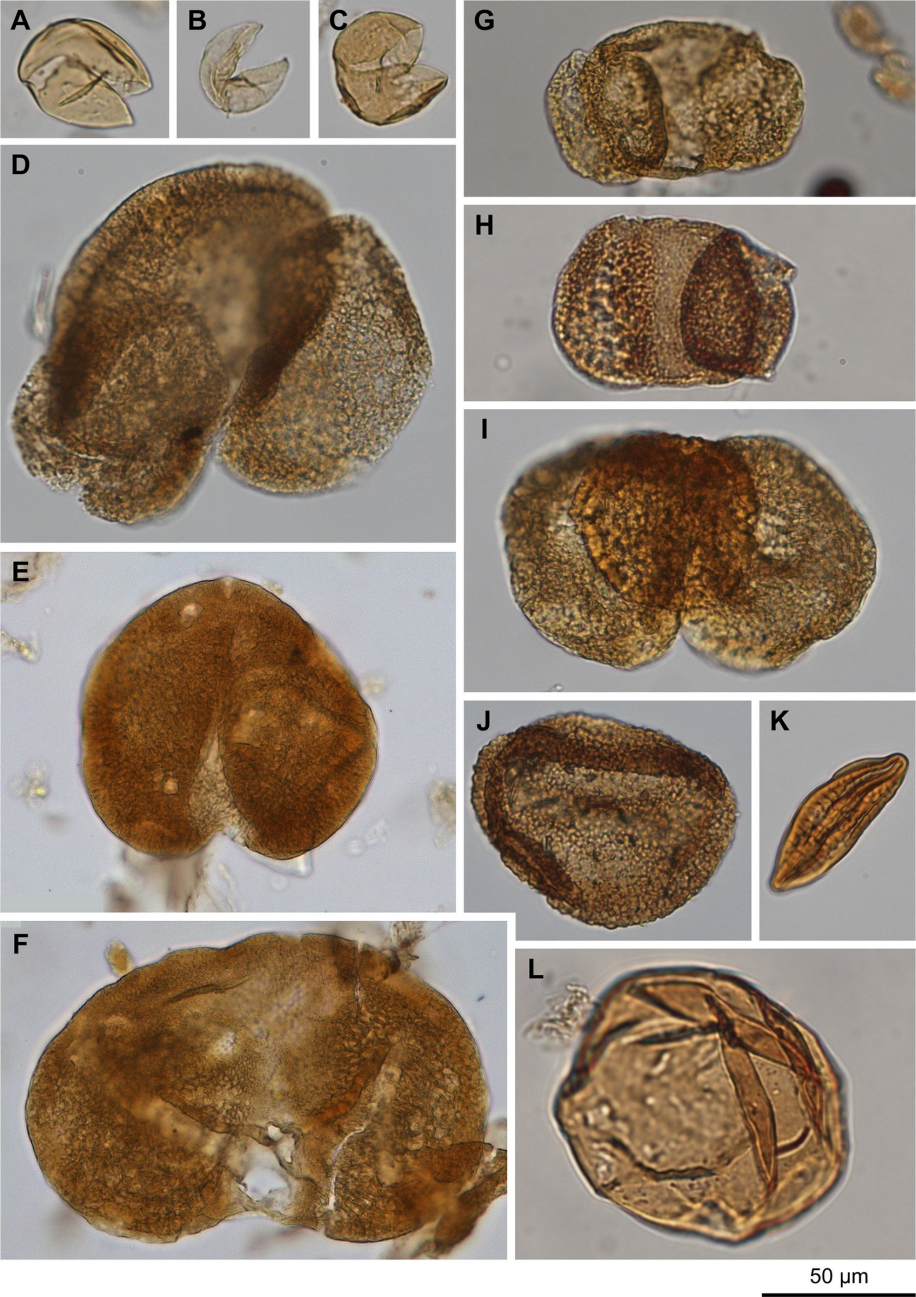
Selected Acrogymnospermae pollen types. (A-C) Cupressaceae/Taxaceae undiff., (D) *Abies*, (E) *Cedrus*, (F) *Picea*, (G, H) *Pinus* undiff., (I) Podocarpaceae/*Cathaya*-type, (J) *Tsuga*, (K) *Ephedra viridis*-type, (L) *Larix*/*Pseudotsuga*.

**Fig 5:**
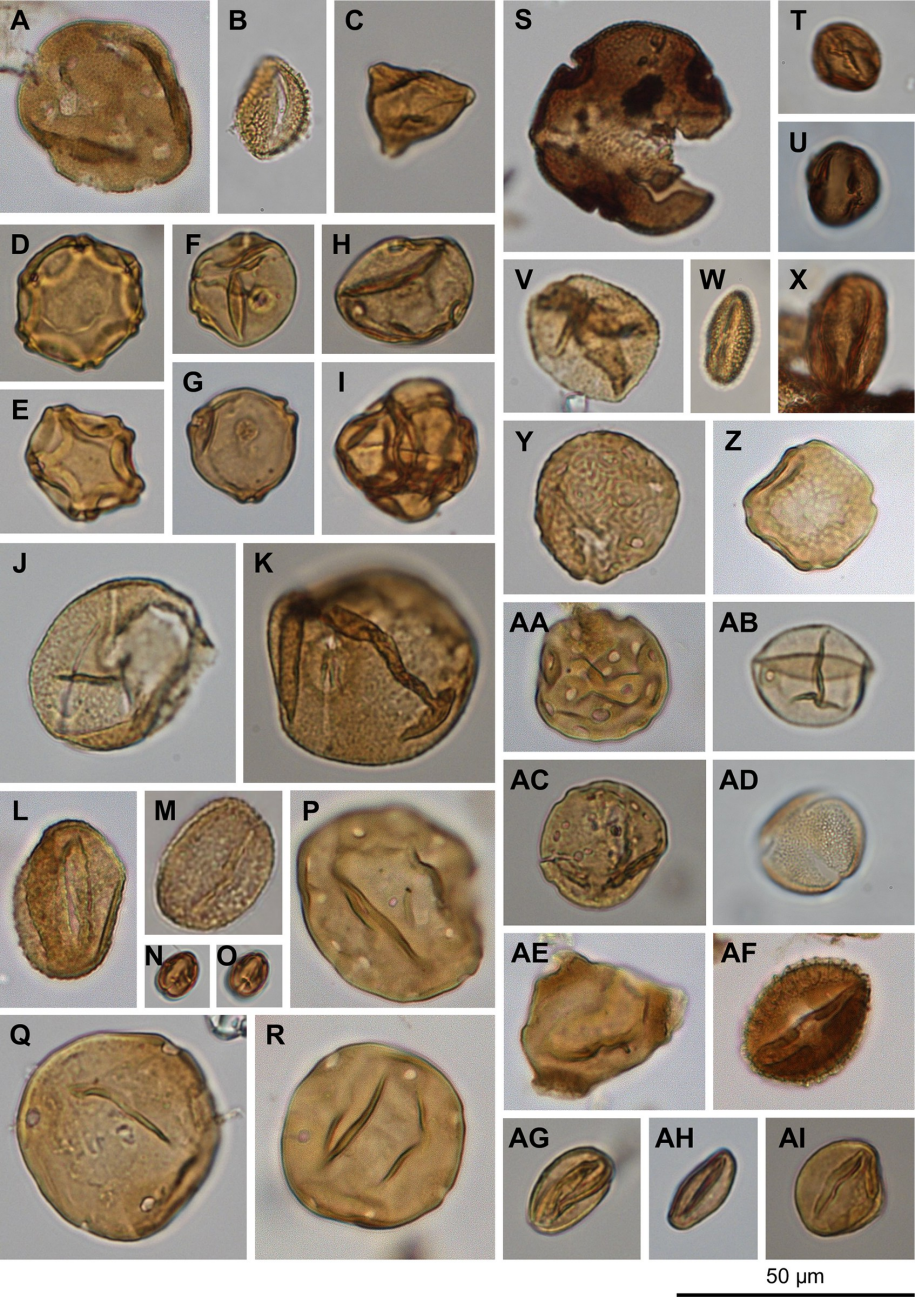
Selected Angiospermae pollen types. (A) *Liquidambar*, (B) *Ilex*, (C) Elaeagnaceae/*Shepherdia argentea*-type, (D, E) *Alnus*, (F, G) *Betula*, (H) *Celtis*, (I) Ericaceae, (J, K) *Fagus*, (L, M) *Quercus*, (N, O) *Castanea*/*Lithocarpus*, (P) *Juglans*, (Q) *Carya*, (R) *Pterocarya*, (S) *Tilia*, (T, U) cf. Rosaceae, (V) cf. *Populus*, (W) *Salix*, (X) *Acer*, (Y, Z) *Ulmus*/*Zelkova*, (AA) *Sarcobatus*, (AB) Poaceae, (AC) Amaranthaceae, (AD) cf. Brassicaceae, (AE) unknown? Onagraceae, (AF) unknown tricolporate, reticulate, (AG-AI) unknown tricolpate, psilate.

**Fig 6:**
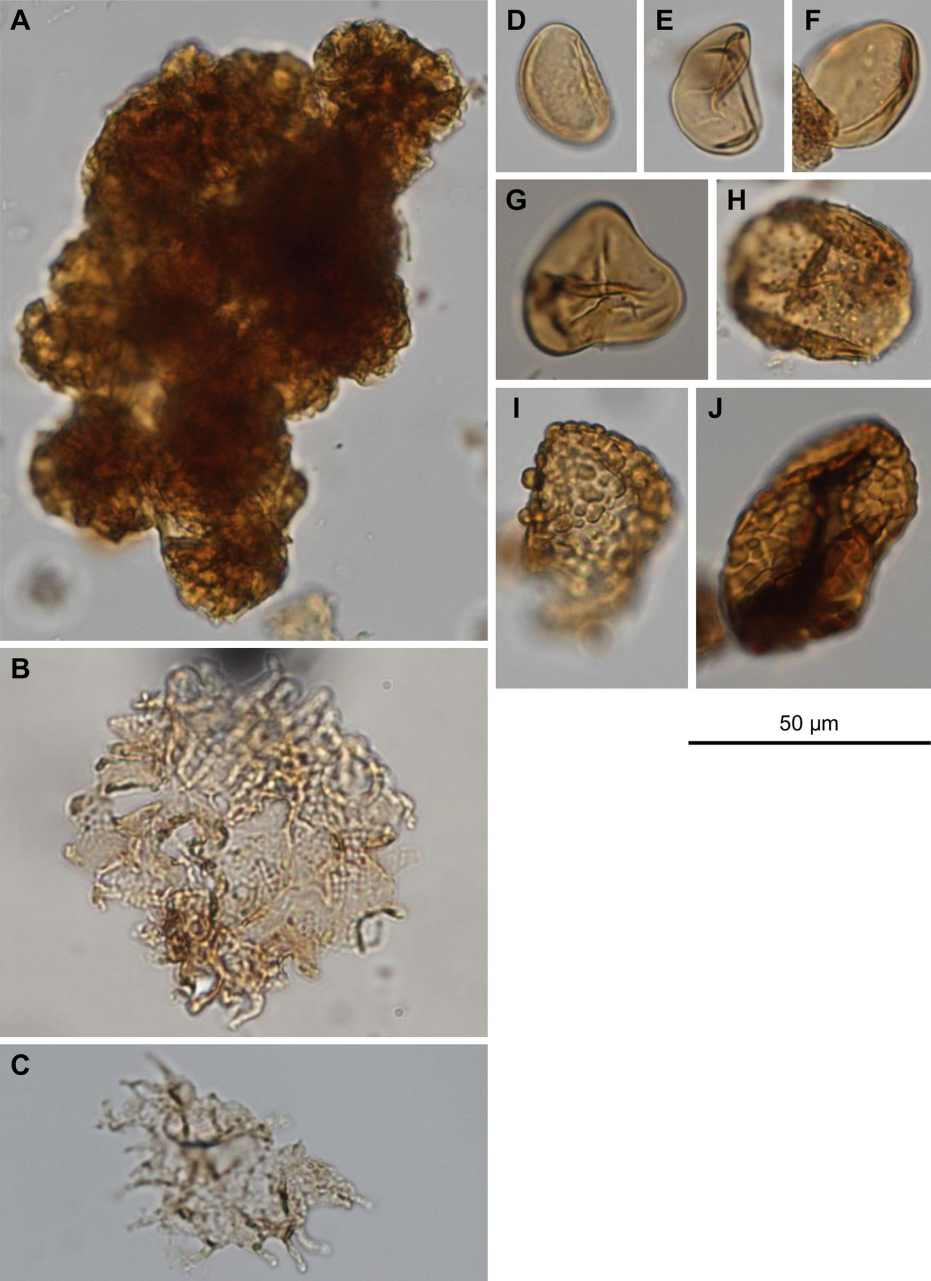
Selected non-pollen palynomorphs. (A) *Botryococcus*, (B, C) *Pediastrum*, (D-F) monolete, psilate Polypodiophyta unk., (G) trilete Polypodiophyta unk., (H) cf. *Osmunda*, (I, J) monolete, coarsely verrucate Polypodiophyta unk.

**Fig 7:**
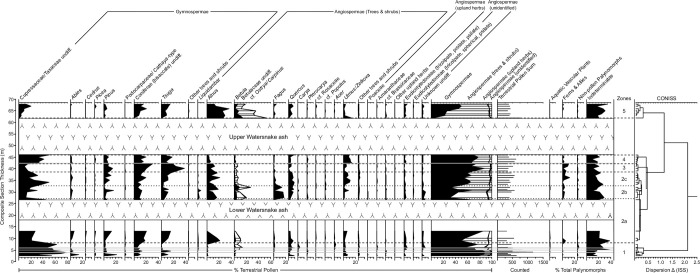
Percentage diagrams of major (>1%) pollen types and spores. Plotted zonation, used in discussion of pollen data, is supported by CONISS dendrogram constructed with percentage data from terrestrial pollen. Ashes are plotted, with thin airfall ashes (gray lines) occurring in zones 1 and 4.

**Fig 8:**
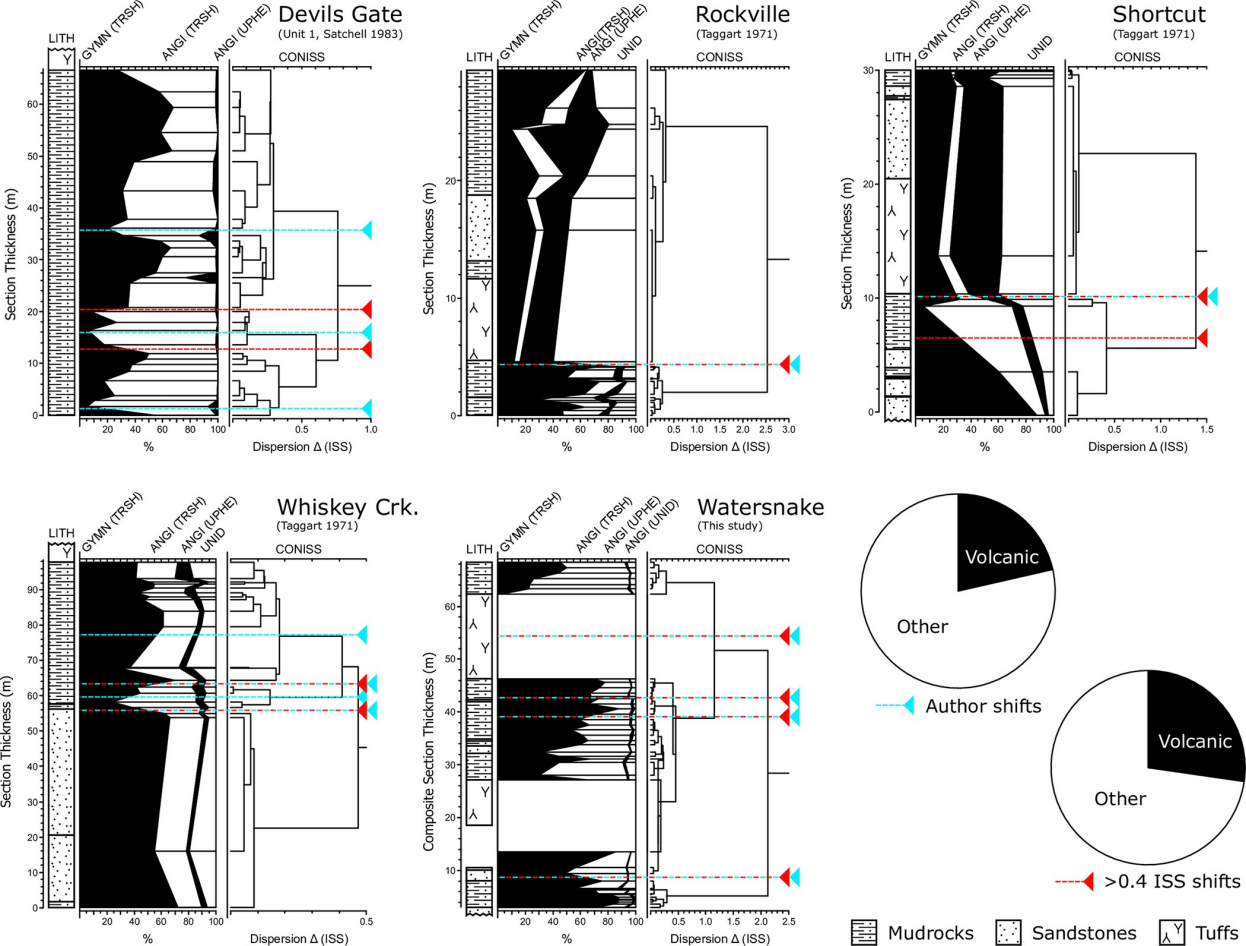
Summaries of stratigraphic and pollen data from the Sucker Creek Formation. Pollen data are broken into gymnosperm (GYMN), angiosperm (ANGI), unidentified (UNID), arboreal (TRSH), and/or nonarboreal (UPHE) components. Major shifts in pollen spectra where qualitatively indicated by the original author (blue) and quantitatively indicated by an increase in dispersion (increase in sum of squares) between CONISS clusters greater than 0.4 are plotted. 3 of 13 author-indicated shifts and 3 of 11 of CONISS-indicated shifts correlate with the deposition of thick tuffs.

Pollen was extracted from 59 samples (Figs 4–6), of which 12 were excluded due to poor pollen preservation (identifiable grains < indeterminable grains). CONISS separated pollen spectra into 5 distinct zones (Fig 7). Of the four shifts between zones identified, one co-occurs with the “upper Watersnake ash” (Fig 8). Phytolith extraction from 12 samples yielded only one sample (45.75 m composite section) that was well enough preserved to warrant counting. In that sample, phytolith preservation was moderately good but the biosilica assemblage as a whole was affected by substantial secondary silicification. Two phytolith samples (61.75, 65.75 m composite section), that had only poorly preserved phytoliths and diatoms with altered texture and abundant secondary silica (S1 Fig), were studied in a semi-quantitative fashion (S4 Table). The remainder of the samples contained no recognizable biosilica.

## WS Zone 1 (2.4–7.9 m composite section)

Cupressaceae/Taxaceae pollen dominated the spectra at the base of the section (22–78%), with lesser abundances of other conifers including *Abies* (0–14%), *Pinus* (1–22%), *Tsuga* (<1%–15%), and undifferentiated bisaccate conifers (3–13%). Angiosperm pollen was relatively rare through the zone, with *Betula* (1–13% hereinafter including undifferentiated Betulaceae), *Quercus* (1–10%), *Ulmus*/*Zelkova* (1–7%), and an unknown tricolpate, prolate type (0–6%) being the most common. Herbaceous pollen, aquatic pollen, and fern spores were nearly absent. Three thin (≤2 cm thick) ashes occur at 3.87, 4.93, and 5.78 m composite section within the zone, but did not appear to have had any significant impact on pollen spectra. No phytolith assemblages were recovered from this interval.

The macrofossil collection included 853 specimens assigned to a morphotype: 476 non-monocot angiosperm foliage, 311 conifer foliage, 63 reproductive structures, and 3 *Equisetum* stems (S5 Table). These specimens were assigned to 37 total morphotypes, 24 of which were foliar morphotypes and 19 were non-monocot angiosperm foliar morphotypes. The fossil flora was of low evenness, with *Quercus simulata* and *Glyptostrobus oregonensis* together accounting for 79% of all foliage specimens collected, and *Q*. *simulata* itself accounting for 73% of non-monocot angiosperm foliage. Other notable foliar morphotypes include those attributed to the following genera (in order of decreasing abundance): *Platanus*, *Betula*, *Fraxinus*(?), *Arbutus*(?), *Pinus*, *Acer*, *Ulmus*, *Fagus*, and *Abies*. Notable reproductive structures include *Glyptostrobus oregonensis* cones, winged seeds of *Abies* and *Pinus*, samaras of *Acer*, *Fraxinus*, *Ptelea*(?), and *Betula*/*Alnus*, the reproductive bracts of *Tilia*, and an endocarp of *Sabia* [71].

## WS Zone 2 (7.9–38.5 m composite section)

This zone was generally marked by an abrupt decline in Cupressaceae/Taxaceae pollen (12–50%), with increases in other conifer types, including *Abies* (<1%–6%), *Pinus* (1–16%), *Tsuga* (2–15%), and undifferentiated bisaccate conifers (5–20%). *Alnus* (1–22%) and other Betulaceae pollen (2–27%) were, on average, more abundant than before. An unattributed monolete, psilate fern spore became increasingly common through this interval (0–12%). A subdivision of this zone lying above the “lower Watersnake” ash, WS Zone 2b (27–32.5 m composite section), is of particular note. *Alnus* (1–16%) and other Betulaceae (3–27%) pollen remained elevated while several other angiosperm tree pollen became abundant for the first time, including *Liquidambar* (0–6%) and *Fagus* (0–17%). No phytolith assemblages were recovered from this interval. Charcoal recovered as float from the base of the “lower Watersnake” ash are attributed to conifers based on the uniform cell size and lack of vessel architecture (S2 Fig), likely *Glyptostrobus oregonensis*, given its abundance in the macrofossil collection.

## WS Zone 3 (38.5–42.1 m composite section)

A shift in conifer pollen composition was the defining feature of WS Zone 3; Cupressaceae/Taxaceae was reduced (9–15%). *Tsuga* (18–38%) and undifferentiated bisaccate conifers (17–31%) reached their highest abundances of the record. Little change is noted in angiosperm pollen types and fern spores through this zone. No phytoliths assemblages were recovered from this interval.

## WS Zone 4 (42.1–45.8 m composite section)

Conifer pollen composition shifted again in WS Zone 4 with a return of high Cupressaceae/Taxaceae pollen abundance (31–50%) at the expense of *Tsuga* (4–17%) and undifferentiated bisaccate conifers (9–19%). *Ulmus* again increased in abundance (9–15%), while *Alnus* (2–6%) and *Betula* (1–2%) decreased. The monolete fern spore was virtually absent in this zone. Despite moderately poor biosilica preservation, ~200 phytoliths were enumerated (93 diagnostic) at 45.75 m composite section. Forest indicator phytoliths comprised 68.5% of diagnostic morphotypes, consisting largely of forms typical of woody dicotyledonous plants (e.g., polygonal epidermal cells, vesicular infillings, sclereids). The grass phytolith assemblage included primarily morphotypes diagnostic of grasses in the Pooideae subfamily (69.0% of GSSCP), as well as several morphotypes typically produced by PACMAD grasses.

## WS Zone 5 (61.7–67.8 m composite section)

The uppermost part of the section, WS Zone 5, occurs above the “upper Watersnake ash” and marked a sharp shift in the pollen spectra. *Alnus* (27%), *Betula* (52%), and *Ulmus* (14%) were the initial dominant components of the zone, but their abundances declined through the section while Cupressaceae/Taxaceae, *Tsuga*, and undifferentiated bisaccate conifer pollen increased in younger layers. Two poorly preserved samples from this zone contained forest indicator phytoliths as well as GSSCP from (primarily) Pooideae.

### Other Succor Creek pollen

Applying the same CONISS threshold to existing pollen data, increase in the sum of squares between clusters (ISS) > 0.4, eleven shifts in vegetation were inferred, compared with thirteen shifts qualitatively assessed by the authors (Fig 8). Of these, three co-occur with tuffs; other mechanisms must be responsible for the remaining eight (based on ISS) or ten (based on original author descriptions) shifts (Fig 8).

## Discussion

### Vegetation of the Watersnake locality of the Succor Creek flora

Paleobotanical evidence points to distinct vegetation types in the lake marginal swamp and in upland forests. Pollen assemblages are dominated by Cupressaceae/Taxaceae and the foliage assemblages by *Glyptostrobus oregonensis* (while other Cupressaceae and Taxaceae macrofossils are rare) and *Quercus simulata*, highlighting the importance of those species in Miocene Watersnake vegetation. *Glyptostrobus* today is represented by a single species, *Glyptostrobus pensilis* (Staunton ex D. Don) K. Koch, with purportedly natural populations growing in lowland swamps, floodplains, and river deltas of southeast China and northern Vietnam [72, 73]. Although extinct species of *Glyptostrobus* lived in a diverse range of climate types through its geologic history, a preference for swamp, floodplain, and river delta environments appears consistent through time [72]. *Quercus simulata* is a conspicuous member of many Miocene fossil floras in the Pacific Northwest (USA), representing foliage with a large range of continuous variation of leaf morphology across and within sites [31, 34, 74]. Its prevalence and abundance in several regional floras, suggests that it was a dense-growing riparian species [74]. *Platanus* foliage is the third highest fossil in abundance (5.4% of total foliage) and is also prevalent in riparian areas today [75]. Other hardwood taxa (e.g., *Betula*, *Fraxinus*, *Acer*, *Ulmus*, *Fagus*, *Tilia*) are typical of temperate mixed deciduous forests in the eastern US, Europe, and eastern Asia [31], and their generally lower abundance suggests they grew at a greater distance from the lake’s edge [76]. Pollen data suggest that Watersnake vegetation beyond the *Glyptostrobus* swamp consisted of a dense upland forest, given the rarity of herbaceous pollen types. Phytolith data from the upper part of the section are also consistent with an overall forested landscape, but show that open-habitat, primarily C_3_, Pooideae grasses lived around the lake or in forest meadows. Compositionally, pollen spectra point to a landscape with conifer (*Pinus*, *Tsuga*) and hardwood components (*Alnus*, *Betula*, *Ulmus*/*Zelkova*). Similar conifer-hardwood mixtures in Succor Creek pollen spectra have historically been attributed to either the widespread presence of mixed conifer-hardwood forests [38], or spatial averaging of different vegetation types across a topographically diverse landscape, whereby ‘montane’ plants (typically conifers) are well represented in lowland palynofloras [14, 33, 34].

We favor the interpretation that the Watersnake locality supported a mixed conifer-hardwood forest (with abundant *Pinus*, *Tsuga*, *Alnus*, *Betula*, *Ulmus*/*Zelkova*) with proximal *Glyptostrobus* swamps [38], rather than distinct forests separated by paleoelevation [14, 33, 34], based on the mixed macroflora combined with plant macrofossil taphonomy (S6 Table). Whereas several important taxa in the Watersnake pollen spectra are known to frequently be overrepresented in palynofloras due to their high pollen production and wide dispersal (e.g., *Pinus*, *Quercus*, [77]), these taxa are also encountered in our macrofloral collection. *Abies klamathensis* fossil foliage attached to stems, with distinctive circular leaf scars, and well-preserved foliage of *Pinus*, supports their presence in local vegetation, as long distance transport from higher elevations for these organs are not expected [78]. Mixed conifer-hardwood forests have analogues in modern East Asian and Eastern North American forests, although, at the stand scale, conifers are commonly restricted to dry slopes [79, 80].

Our age-depth model (Fig 3) suggests that Watersnake section represents ~30 kyr, in agreement with earlier appraisals of Sucker Creek deposition rates. Cross and Taggart [14] previously reasoned that the fossil-bearing lacustrine shales in the Sucker Creek Formation accumulated within thousands to tens of thousands of years based on assumptions of basin fill rates. Thus, changes that occurred through the section capture processes playing our over ecological time spans, similar to the resolution of many Quaternary paleoecology studies.

### Mechanisms of vegetation change

#### Short-term (≤10^4^ years) trends

Within the studied sections of the Sucker Creek Formation, we observed significant changes to vegetation structure and composition that took place on timescales of ≤10^4^ years. This temporal estimation assumes that deposition rates calculated for Watersnake (~500 years/m, Fig 3) apply across the lacustrine deposits of the formation, such that each ~30–90 m palynological section was deposited in ~15–45 kyr. At Watersnake, the most significant, short-term vegetation changes coincide with the deposition of thick ash-flow tuffs. Following the deposition of the lower Watersnake ash-flow tuff, what we interpret as upland and swamp conifer taxa were not well represented and hardwoods (*Liquidambar*, *Alnus*, *Betula*) formed an early-successional community (Fig 7). *Fagus*, which today typically occurs in late successional communities [81, 82], although monospecific stands are known to inhabit recently disturbed areas as well [83], is a large component of the post-disturbance community (Fig 7). After the deposition of the upper Watersnake ash-flow tuff, the upland and swamp conifer taxa were nearly entirely removed and replaced by *Alnus* and *Betula* (Fig 7), today known for their symbioses with nitrogen-fixing microorganisms [75], which dominated the early successional communities. Watersnake vegetation appears to have recovered to pre-disturbance condition quickly. For example, the early successional zone following the lower Watersnake ash-flow tuff (WS zone 2b) lasts only ~3 kyr based on the median ages of our age-depth model, although we acknowledge considerable uncertainty in this estimate (95% confidence interval ± ~50 kyr). The early successional zone above the upper Watersnake ash-flow tuff (WS zone 5), although truncated before fully pre-disturbance conditions returned, continued to change towards the reestablishment of upland and swamp conifer taxa at the expense of nitrogen-fixing hardwoods. In contrast to ash-flow tuffs, thinner airfall ashes appeared to have had little or no ecological impact.

Other, more minor vegetation change within the Watersnake section might be linked to local changes in surface hydrology. In particular, a reduction in *Glyptostrobus* in favor of other conifers (*Tsuga*, *Pinus*, WS zone 3) correlates with the shift from shale to claystone deposition in the middle lacustrine unit (Fig 2). The claystone is relatively enriched in Ti, suggesting enhanced siliciclastic sedimentation relative to shales above and below it. We hypothesize that the *Glyptostrobus* swamp was buffering sediment from the main lake basin and the reduction of the swamp, the cause of which is unknown, enhanced siliciclastic deposition.

**Fig 9:**
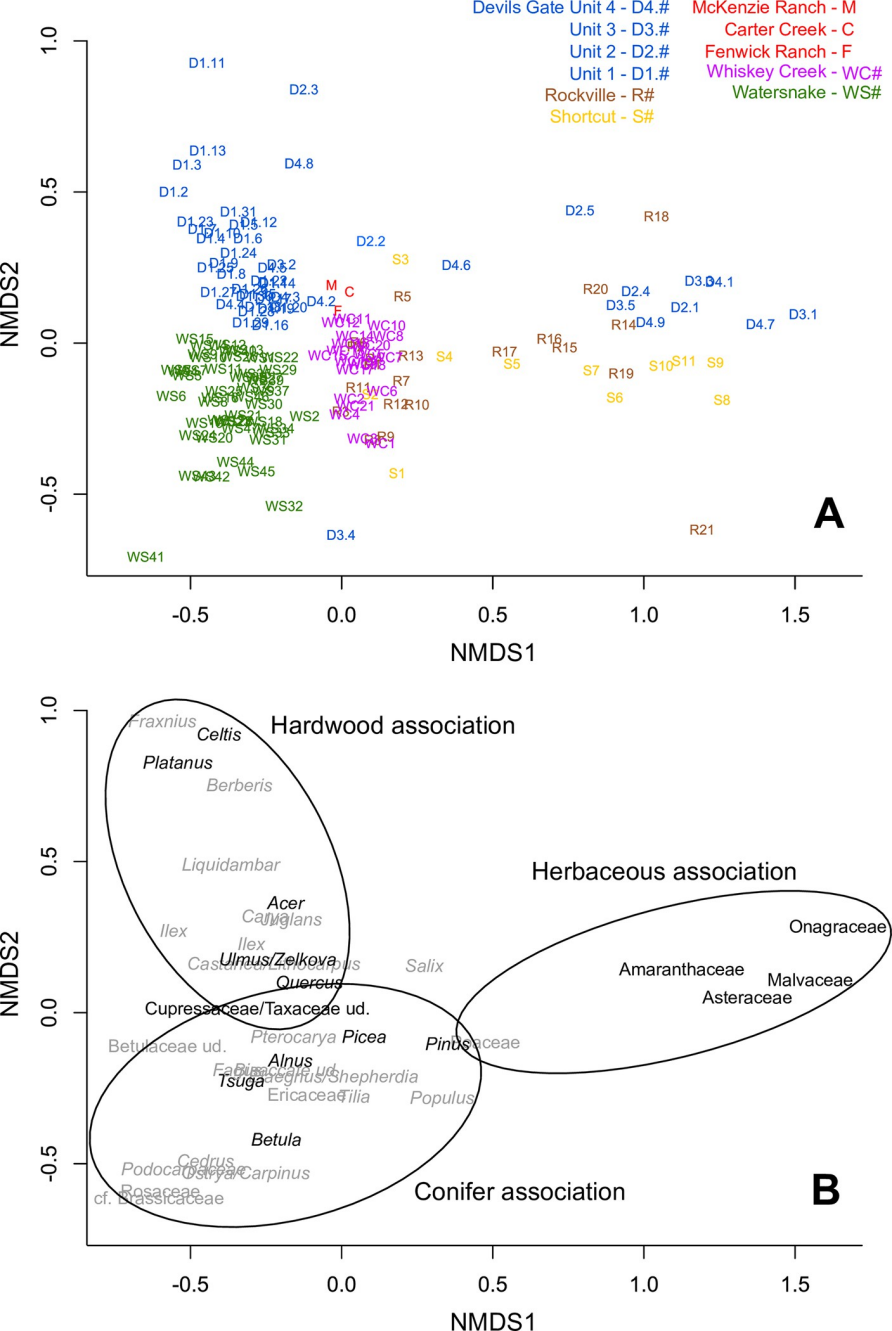
NMDS ordination of Succor Creek pollen spectra. (A) Ordination plotted by sample number, labelled by their locality and their sequential number from the base of the section, except for Devils Gate localities which are additionally labelled by their unit from Satchell [38]. (B) Ordination plotted by pollen type with taxa significant to the discussion in black.

Similar to Watersnake, major vegetation change in the Rockville and Shortcut sections correlates with the deposition of thick tuffs. In both sections, existing mixed forests (*Picea*, *Pinus*, *Alnus*, *Ulmus*/*Zelkova*) were replaced by herbaceous (Amaranthaceae, Asteraceae, Malvaceae) and, later, *Pinus*-dominated communities (Fig 9). Both communities are consistent with the modern disturbance autecology of those herbs and *Pinus*, supporting the traditional interpretation of Rockville and Shortcut vegetation [40]. The duration of early successional communities in the Rockville and Shortcut are unclear due to a dearth of radioisotopic dates for these sections. Other vegetation shifts previously attributed to volcanic disturbance in the Devils Gate (Unit 1), Shortcut, and Whiskey Creek sections [14–16, 38, 40] do not appear to correlate with tuffs or any other stratigraphic evidence of volcanic disturbance (Fig 8). Alternative mechanisms of disturbance, such as fire [14, 38], have yet to be tested.

Overall, palynological data from the Sucker Creek Formation are consistent with the hypothesis that, in several cases, volcanic disturbance drove MCO plant community composition change in the Pacific Northwest over short timescales (≤10^4^ years, *sensu* [14]). Multiple mechanisms of volcanic disturbance are evident within Sucker Creek strata, but pyroclastic flows, preserved as thick ignimbrites, notably resulted in three (of eleven) large vegetation shifts observed in palynological sections. Relatively large vegetation mortality and longer recovery time are consistent with modern observations of thicker ashfalls or pyroclastic flows. For instance, the Pumice Plain of Mt. St. Helens remains chiefly herbaceous or unvegetated decades after the climactic AD 1980 eruption [84]. Areas of the central Oregon Cascade Range, buried under thick ash and lapilli from the 7.6 ka eruption of Mt. Mazama, were permanently altered to *Pinus contorta* forest, whereas less-impacted stands have since recovered to a more regionally typical *Pinus ponderosa* forest [85, 86]. Airfall ashes, resulting in tuffs up to 4 cm thick in Sucker Creek strata, appeared to have no observable impact on vegetation. In modern ecological studies, plant mortality is low and recovery fast in comparable scenarios where thin ashfall occurred [87], a result echoed by studies of thin ashfalls in Quaternary studies [88–90].

## Long-term (>10^4^ years) trends

Significant differences in vegetation are observed between the studied sections of the Sucker Creek Formation which cannot be attributed to volcanic disturbance. Our geochronological data document that disparate pollen spectra from each locality represent a series of ~15–45 kyr snapshots through an approximately 1 Myr interval of the Middle Miocene (~14.8 to ~15.8 Ma). Our NMDS analysis of available pollen spectra reveals a clear pattern where the vegetation of the Succor Creek flora can be effectively split into three (groups of) snapshots, Rockville/Shortcut, Watersnake, and Devils Gate (Fig 9), independent of inferred successional stage. Discussion here will focus on comparisons to the Watersnake locality. In each case, we will first consider the hypothetical late-successional vegetation, excluding strata immediately above thick ignimbrites, followed by potential early-successional vegetation.

The oldest snapshot is typified by the Rockville locality (~15.78 Ma), which has long been correlated with the Shortcut locality on the basis of lithological and palynological similarities [34, 40]. Although the stratigraphic relationships have never been resolved in this region of the Sucker Creek Formation, we will include the Shortcut, Whiskey Creek, McKenzie Ranch, Carter Creek, and Fenwick Ranch localities within this snapshot based on their geographic proximity (Fig 1) and broad palynological similarity (Fig 9) to the Rockville locality. Taggart originally [40] and in subsequent discussions [14, 15, 68], considered conifer and hardwood components as separate plant associations: *Glyptostrobus* dominated swamp associations; a bottomland/slope association mostly represented by hardwood taxa; a montane conifer association consisting of cool-adapted conifers, chiefly *Picea*, but also *Abies* and *Tsuga*; a *Pinus* association to acknowledge that *Pinus* could be present within several associations; a xeric, mostly herbaceous association [14, 40]. However, note that macrofossils from nearly all of the arboreal components of the vegetation—conifer and hardwood—are found in close stratigraphic association in a comparison of macrofossil [33] and pollen [40] collections at the Whiskey Creek locality. Therefore, similar to Watersnake, we interpret the late-successional vegetation of Rockville-Shortcut as having consisted of a mixed conifer-hardwood forest (with extensive *Glyptostrobus* swamp) rather than representing several elevational vegetation zones. The forests of the Rockville-Shortcut snapshot were likely more cold-adapted than at Watersnake given the abundance of *Picea* (pollen and macrofossils), which today is a characteristic conifer of Northern Hemisphere sub-alpine, montane, and boreal forests [91]. This distinction caused Wolfe [92] to consider the Rockville group as a paleoflora separate from Succor Creek (a “Rockville flora”) and erroneously assign them to the Late Miocene.

The Devils Gate sections consist of at least three snapshots ranging from >15.1 to <14.8 Ma, based on U-Pb geochronology. We now recognize that Satchell’s ‘unit II’, is older than 15.1 Ma, due to its stratigraphic position below the Loggers Ash. ‘Unit III’ ranges from 15.1 to 14.8 Ma, bounded at the bottom and top by the 15.1 Ma Loggers ash and the 14.8 Ma Obliterator Ash, although the pollen-bearing portions of the unit are near the base. ‘Unit I’, with its high-resolution palynological record, is bounded at the top by the 14.8 Ma Obliterator Ash, which also forms an approximate lower bound of ‘unit IV’. Agreeing with Satchell’s original [38] characterization, we interpret the younger units ‘I’ and ‘IV’ as a chiefly late-successional, mixed conifer-hardwood forest, based on the cooccurrence of conifer and hardwood pollen and macrofossils, with an extensive *Glyptostrobus* swamp. Units ‘I’ and ‘IV’ are both characterized by frequent vegetation change. ‘Unit I’ is typified by the increase of Cupressaceae/Taxaceae pollen through time, interpreted as a proliferation of *Glypstostrobus* swamp, and ‘unit IV’ includes intervals in which herbaceous taxa (Amaranthaceae, Asteraceae) dominate, suggesting more open-canopy vegetation; these communities are similar in composition to herb-dominated palynofloras at Rockville-Shortcut (Fig 9). The isolated pollen spectra of the older units ‘II’ and ‘III’ are inferred to have been mostly open, but highly variable, with a dominant herbaceous component (Amaranthaceae, Asteraceae, Onagraceae); spectra rich in hardwoods (alternately *Alnus*, *Quercus*, *Ulmus*/*Zelkova*) and conifers (*Abies*, *Picea*, *Pinus*, *Tsuga*) are present as well. These communities were originally interpreted to be early successional communities [38], and their compositions are strikingly similar to inferred early successional communities at Rockville and Shortcut (Fig 9). The plant associations of the Devils Gate mixed conifer-hardwood forests primarily differ from Watersnake based on the higher abundance and composition of the hardwood component as well as the conifers represented. Whereas Devils Gate sediments are abundant in *Celtis*, *Platanus*, *Acer*, *and Ulmus*/*Zelkova*, Watersnake is rich in Betulaceae, chiefly *Alnus* and *Betula* (Fig 9). Certain conifers today linked to montane habitations are more abundant at Devils Gate compared with Watersnake (*Abies*, *Picea*).

**Fig 10:**
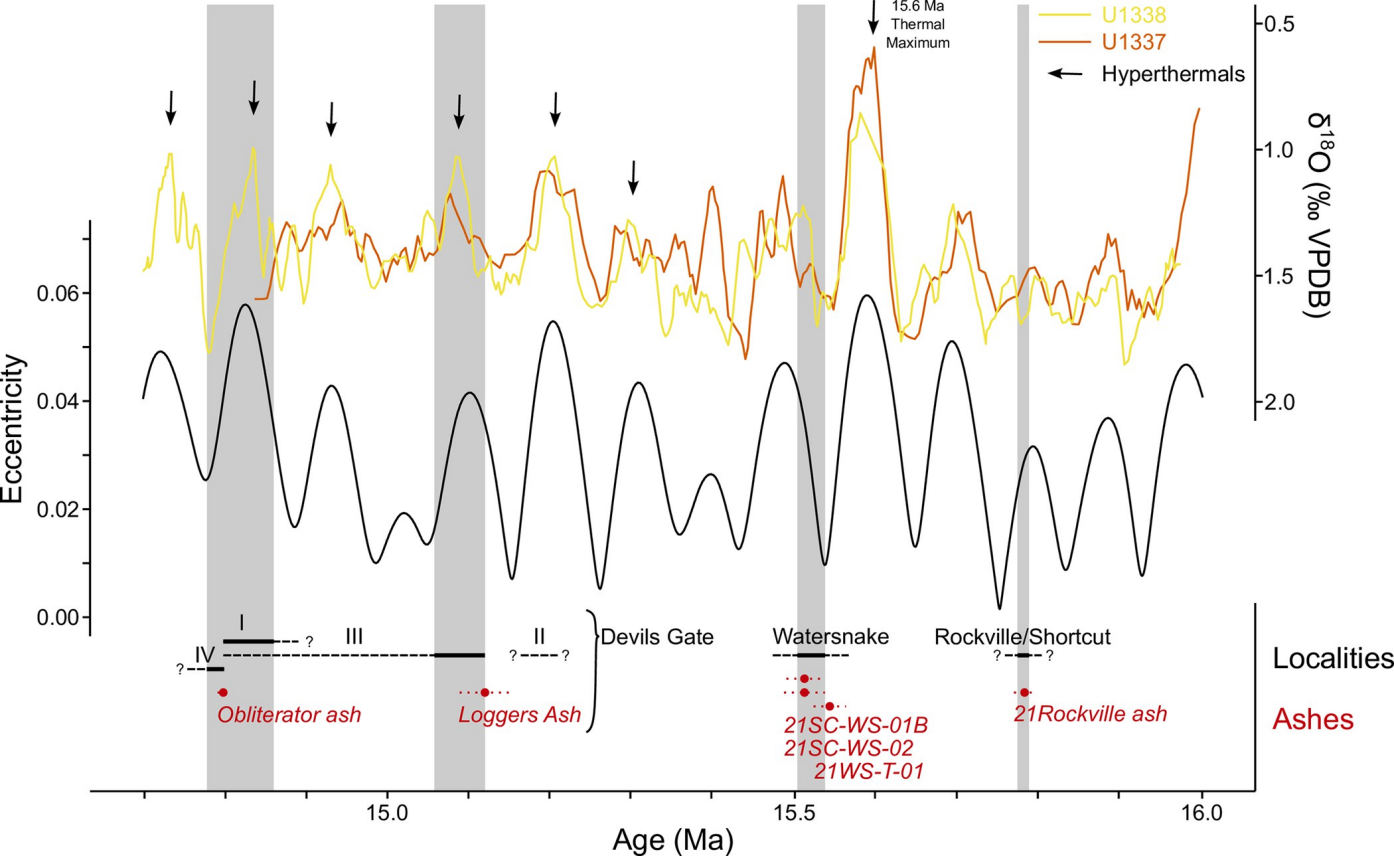
Timing of Sucker Creek stratigraphic palynological sections relative to climate and eccentricity reconstructions. Eccentricity quantities based on Laskar [93] solution. Tropical, eastern Pacific benthic foraminifera δ^18^O records from Integrated Ocean Drilling Program Sites U1337 [94] and U1338 [7] smoothed with a 5 observation rolling mean to emphasize long-term trends. Devils Gate unit 1 distinctly occurs during a cooling interval. Temporal extent of pollen-bearing sections (black lines with thin black lines showing uncertainty) and individual U-Pb dates (red dots are median probability ages, red dotted lines show uncertainty) are plotted.

Differences between the Succor Creek snapshots were previously recognized by Fields [34], who, assuming that Succor Creek localities were roughly contemporaneous, suggested that these vegetation differences were due to a N–S elevational gradient. In this model, more southerly localities (e.g., Whiskey Creek) were richer in upland conifers due to higher paleoelevation. The new U-Pb chronology presented here suggests that floral localities in the Sucker Creek Formation were deposited intermittently over ~1 Myr, opening up the possibility that differences in floral composition between localities are instead linked to long-term climate changes. Middle Miocene climate strongly varied with periodicity matching 100 and 400 kyr eccentricity cycles [6, 7], resulting in periodic “transient hyperthermal events” from ~15.7 to ~14.7 Ma [11]. The Rockville snapshot (~15.8 Ma), despite occurring during an eccentricity maximum, co-occurs with relatively cool interval in the δ^18^O records and predates the first “hyperthermal event” (Fig 10). Watersnake (~15.5 Ma) was deposited during an eccentricity-paced warming trend, which was modestly warmer than Rockville. The Devils Gate sections (>15.1 to <14.8 Ma) tended to be deposited during eccentricity maxima which were warmer than the Rockville or Watersnake intervals. Although elevation may also have played a role, these snapshots reveal that the cool-adapted, mixed forests of Rockville-Shortcut localities, the warm-adapted, mixed forests of Watersnake, and the diverse, mixed forests of Devils Gate (~15.1–14.8 Ma) existed in distinct climate spaces on eccentricity timescales. Devils Gate “Unit 1” also co-occurred with an eccentricity-paced cooling interval (Fig 10), providing an explanation for the gradual proliferation of *Glyptostrobus* swamp through that section, if hydroclimatic changes accompanied the cooling.

In contrast to previous authors, we propose that climate change drove the differences between Succor Creek sections and find no evidence that volcanic disturbance was so frequent and severe on long timescales (>10^4^ years) as to overprint this signal. Ebinghaus et al. [17] suggested that climate trends were not observable in their Columbia Plateau palynological data due to the frequency of volcanic disturbance on the Miocene landscape. Specifically, that study invoked explosive volcanic disturbance from the Snake River Plain frequently impacting Columbia Plateau vegetation. The Succor Creek flora, directly adjacent to the contemporaneous Lake Owyhee Volcanic Field, preserves several floral assemblages indicative of late successional, dense, mixed forests and swamps and evidence of vegetation change not tied to any volcanic deposit. We contend that persistent early- and mid-successional Miocene plant communities documented on the Columbia Plateau [17] were the product of to slow soil formation on lava flows rather than frequent, explosive volcanic disturbance.

### Implications for Cenozoic vegetation and climate reconstructions

Palynological data presented here suggest that the approach taken in many global syntheses of paleobotanical data ignores variation in plant communities and may misdiagnose plant communities and biomes. Most pre-Quaternary Cenozoic palynological syntheses, on global [5, 95], continental [96], and regional [97, 98] scales, use palynofloras as discrete points in long spatiotemporal frameworks. Global syntheses have provided a robust test of global Cenozoic climate models; however, they suffer from the condensation of shorter-term variation and time-averaging across localities. Such syntheses include large numbers of roughly contemporaneous floras filtered by geochronologic age, which are used to interpret the palynological data at the biome level. This approach assumes that each flora’s composition and structure will have a central spatiotemporal tendency in equilibrium with prevailing Cenozoic climate trends and regional physiography. Regional approaches provide a more granular spatial view of floral composition and structure, both because the inherently smaller number of localities means that binning to infer floral composition and structure is less frequently done. Most regional studies interpret palynological data at the community level. Practices at both the global/continental (biome) and regional (community) scales seek to assign a central tendency to flora composition and structure, usually ignoring the variance around that average—driven by limited available palynological data, a lack of stratigraphic control, and a lack of high-precision age control.

Quaternary paleoecological studies have used a different approach focused on the variability of biomes and communities in response processes that play out over ecological time scales. These studies demonstrate that plant associations do not maintain consistent composition or structure for more than 2000–3000 years [99, 100]. The largest syntheses of Quaternary pollen data, continental isopoll maps and related pollen-informed models which synthesize pollen spectra produced from hundreds of lake sediment records, broadly concur with this view. They suggest that few plant associations are maintained in place for longer than 6 kyr or so [101–104] due to the individualistic response of species to precession-driven postglacial climate change [105], but also accelerating human land use [106]. Edaphic controls can produce relatively stable vegetation through the Holocene, such as the *Pinus*-Cupressaceae-*Quercus* forests on ultramafic soils of the Klamath Mountains and the *Pinus contorta* forests of the rhyolitic Yellowstone Plateau. Even in these cases, forest structure changes as a function of climate variability [107, 108].

Indeed, stratigraphic palynology from the Sucker Creek Formation demonstrates plant association change due to climate change, disturbance, and other mechanisms on multiple timescales, reminiscent in frequency and magnitude of vegetation changes observed in Quaternary records. These dramatic changes distinctly cool-temperate conifer forests (abundant *Picea* pollen), early-successional herbaceous associations, and cool-temperate mixed forests (abundant *Alnus*, *Celtis*, *Carya*, etc.) have long been recognized [14]. Attempting to characterize a central tendency to the Succor Creek flora without a large, systematic set of pollen spectra would result in mischaracterizations of the community, biome, and prevailing climate. The unlucky selection of samples through intervals of volcanic disturbance or hydrologic change could lead to a mischaracterization of the central tendency of the Succor Creek flora [109]. An herbaceous assemblage, here recognized as early successional, might be interpreted as an arid or semi-arid steppe, or isolated spectra rich in *Picea* or *Tsuga* might cause the entire flora to be interpreted as cool-adapted and montane in aspect. Writing about Pacific Northwest floras and the Succor Creek flora in particular, Wolfe [91 p. 88] observed, “…interpretation of a single [pollen] spectrum can be highly misleading; in a region of active volcanism, for example, one major eruption could drastically change the regional vegetation and hence pollen rain.”

Our work suggests that such variation within fossils floras should be explicitly considered when attempting to generalize a biome or community signal in a global synthetic study. The amount of short-term “noise” present in current global/continental and regional palynofloral syntheses relative to the signal of prevailing Cenozoic climate is unclear and stratigraphic palynology studies with sufficient resolution and stratigraphic extent to test for short-term variability are rare (e.g., [60, 110]). However, the noise of short-term variation is probably especially high in the Neogene of the Pacific Northwest, where most pollen-bearing sequences contain abundant volcanic deposits and many of the sedimentary basins themselves are thought to be volcanic in origin [19, 111, 112]. Recognition of short-term variability then becomes critical in the assessment of long-term vegetation and climate trends—short-term variability that likely characterized most plant communities and biomes of the past.

## Supporting information

S1 Table Existing radioisotopic age controls in the Sucker Creek Formation. (XLSX)

S2 Table XRF standards. (XLSX)

S3 Table CA-TIMS U-Pb isotopic data for volcanic tuff in the Sucker Creek Formation. (XLSX)

S4 Table Phytolith morphotype assemblage from the Watersnake locality, Succor Creek flora. (XLSX)

S5 Table Macrofossil assemblage from the Watersnake locality, Succor Creek flora. (XLSX)

S6 Table Floral list from the Watersnake locality, Succor Creek flora. (XLSX)

S1 Fig Selected biosilica bodies from the Watersnake locality, Succor Creek flora. (A) Diatom, regular preservation (thin, transparent walls, no filling), (B) Diatoms, frustules filled to varying degrees with secondary silica, (C) Presumed filling (secondary silica) of equidimensional diatom, superficially similar to, e.g., palm phytolith (Spheroid echinate), (D) Forest indicator phytolith morphotype (Spheroid ornate), (E) Bilobate or Crenate Grass Silica Short Cell Phytolith (GSSCP) fragment, (F) Crenate GSSCP, diagnostic of Pooideae.
(TIF)

S2 Fig Charcoal macrofossil from the Watersnake locality, Succor Creek flora. (A) Charcoal macrofossil. Thin sections of the same in radial (B) and transverse (C) section. The lack of vessel architecture in (C) is indicative of conifer wood, although finer taxonomic identification is difficult due to charcoalification.
(TIF)
