# Supplementary figures and images for: Mechanisms of rapid plant community change from the Miocene Succor Creek flora, Oregon and Idaho (USA)
Source: PLoS One. 2024 Nov 8;19(11):e0312104. doi: 10.1371/journal.pone.0312104 (PMC11548735; doi:10.1371/journal.pone.0312104)

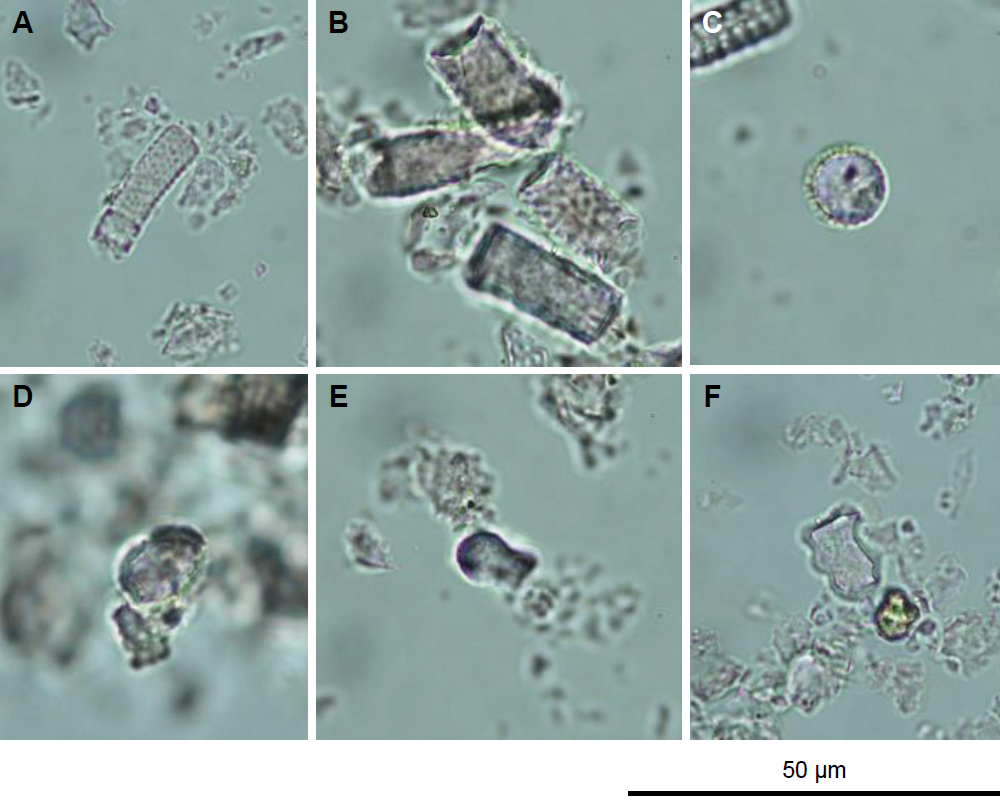

Supplement: S1 Fig — (A) Diatom, regular preservation (thin, transparent walls, no filling), (B) Diatoms, frustules filled to varying degrees with secondary silica, (C) Presumed filling (secondary silica) of equidimensional diatom, superficially similar to, e.g., palm phytolith (Spheroid echinate), (D) Forest indicator phytolith morphotype (Spheroid ornate), (E) Bilobate or Crenate Grass Silica Short Cell Phytolith (GSSCP) fragment, (F) Crenate GSSCP, diagnostic of Pooideae. (TIF) [file pone.0312104.s007.tif]

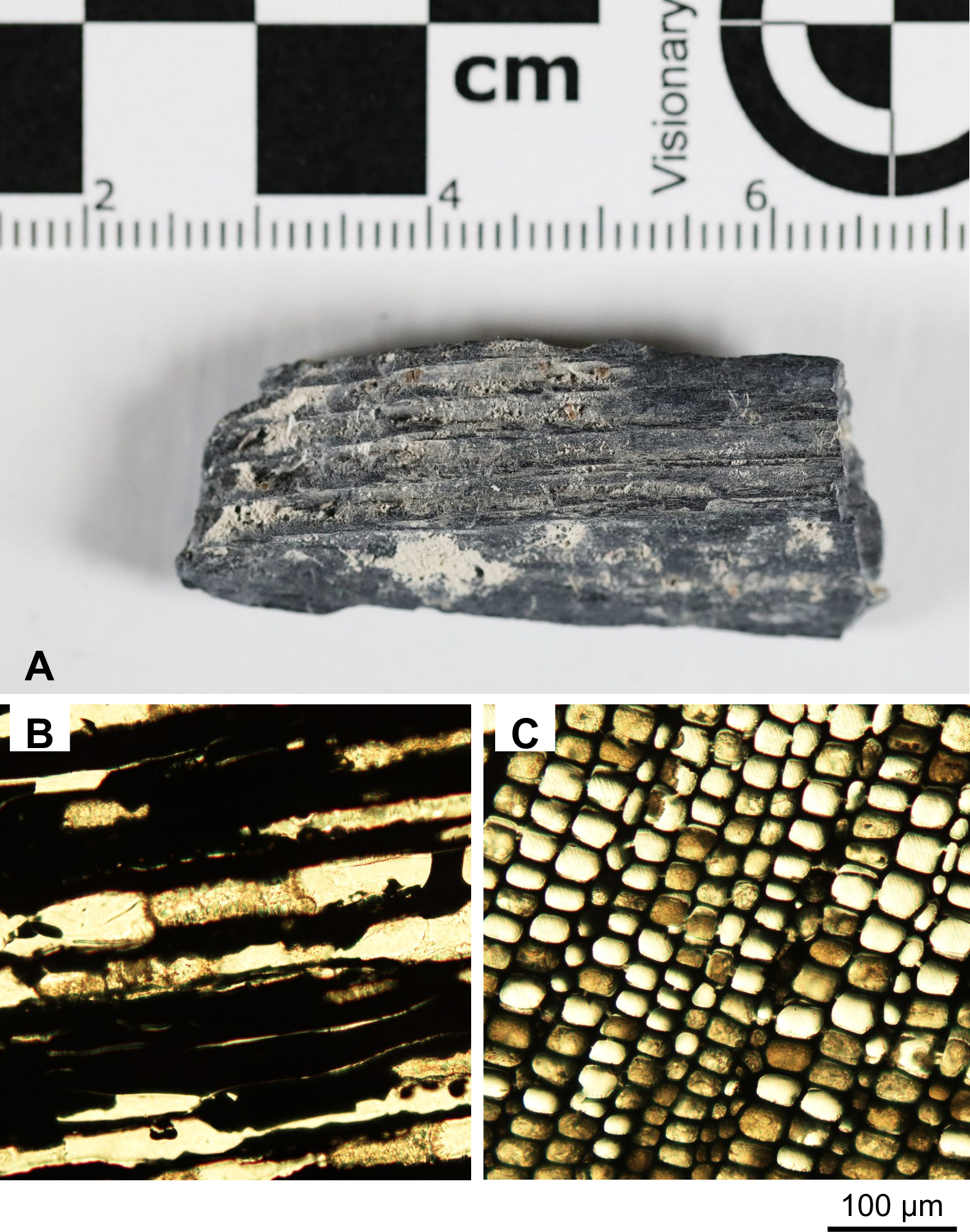

Supplement: S2 Fig — (A) Charcoal macrofossil. Thin sections of the same in radial (B) and transverse (C) section. The lack of vessel architecture in (C) is indicative of conifer wood, although finer taxonomic identification is difficult due to charcoalification. (TIF) [file pone.0312104.s008.tif]
